# Discovery of insect *Blaps rhynchopetera* Fairmaire extracts with anti-tyrosinase activity and anti-melanin deposition

**DOI:** 10.3389/fphar.2025.1595534

**Published:** 2025-08-20

**Authors:** Lamei Zhang, Kaixun Cao, Xizhe Yang, Shengwen Zhou, James Mwangi, Chengye Wang, Yifan Chen, Chengchen Zhang, Ziyi Wang, Min Zhao, Lei Shi, Qiumin Lu

**Affiliations:** ^1^ Yunnan Key Laboratory of Breeding and Utilization of Resource Insects, Key Laboratory of Breeding and Utilization of Resource Insects of National Forestry and Grassland Administration, Institute of Highland Forest Science, Chinese Academy of Forestry, Kunming, China; ^2^ College of Life Sciences, Nanjing Agricultural University, Nanjing, China; ^3^ Engineering Laboratory of Peptides of Chinese Academy of Sciences, Key Laboratory of Bioactive Peptides of Yunnan Province, KIZ-CUHK Joint Laboratory of Bioresources and Molecular Research in Common Diseases, National Resource Center for Non-Human Primates, National Research Facility for Phenotypic and Genetic Analysis of Model Animals (Primate Facility), and Sino-African Joint Research Center, New Cornerstone Science Laboratory, Kunming Institute of Zoology, The Chinese Academy of Sciences, Kunming, Yunnan, China; ^4^ Kunming College of Life Science, University of Chinese Academy of Sciences, Beijing, China

**Keywords:** *Blaps rhynchopetera* Fairmaire, TYR inhibitory, cosmetics, network pharmacology, molecular docking

## Abstract

**Background:**

*Blaps rhynchopetera* Fairmaire is a medicinal insect that has been used for a long time by minority ethnic groups in Yunnan, China, due to its immunomodulatory function. However, its potential applications in cosmetics have not been reported.

**Methods:**

*In vitro* experiments were used to verify whether the extracts of *B. rhynchopetera* Fairmaire (EBR) have the effect of inhibiting TYR activity and eliminating melanin deposition. Subsequently, *in vivo* experiments were conducted to verify further the potential application of the EBR in whitening. To explore the whitening components of the EBR, we employed UHPLC-Q-TOF MS to identify the compounds present in the EBR and utilized network pharmacology to compare them with the genes involved in whitening in the database, thereby obtaining the intersection of compound targets and whitening targets. Then, protein-protein interaction network analysis, gene ontology enrichment and Kyoto Encyclopedia of Genes and Genomes pathway enrichment analysis were carried out to explore the main components of the whitening effect of the EBR. Further, molecular docking and molecular dynamics simulation were used to verify the correlation between the main components of the EBR and the essential target molecules.

**Results:**

*In vitro* and *in vivo* studies have found that the EBR exhibits anti-oxidation, TYR-inhibiting activity, and the elimination of melanin deposition. Subsequently, 1538 metabolites were identified by UHPLC-Q-TOF MS analysis, indicating that EBR is a rich source of bioactive compounds. Among them, 82 kinds of phenols and flavonoids may be derived from external enrichment or self-synthesis. Network pharmacology revealed 41 genes related to pigmentation and TYR inhibition. Molecular docking and molecular dynamics simulation confirmed that myricetin, luteolin, apigenin and quercetin have a high binding affinity with TYR, which may regulate melanin production by inhibiting TYR activity.

**Conclusion:**

In this study, we identified the primary whitening active ingredients of *B. rhynchopetera Fairmaire* and utilized network pharmacology and molecular dynamics simulation to investigate the mechanism underlying the whitening effect of EBR. This work reveals that insect extraction may have broad application prospects in the preparation of cosmetics. To our knowledge, this is the first report identifying the flavonoids quercetin, luteolin, myricetin, and apigenin as whitening-active components in insects.

## 1 Background

Human skin is divided into two main layers: the epidermis and the dermis. The epidermis, from innermost to outermost, consists of the stratum corneum, stratum granulosum, stratum spinosum, and stratum basale (Hendriks et al., 2006). Some melanocytes produce melanin in the stratum basale. Melanocytes play a key role in skin and hair pigmentation by producing melanin (Slominski et al., 2005; Yamaguchi et al., 2007). The formation of melanin is a complex multi-reaction process that involves the hydroxylation of tyrosine, followed by further oxidation and polymerization of oxidative metabolites to produce macromolecular phenolic polymers. Skin pigment production is affected by several variables, including UV, blue light from the sun, inflammation, air pollution, hormone levels, genetics, tumors, and other medications (Goelzer Neto et al., 2022). In addition to absorbing UV and visible light, melanin also scavenges free radicals and antioxidants, limiting the effects of UV on cell macromolecules and shielding cells from toxic damage (Swope and Abdel-Malek, 2018). This protects skin from DNA damage and pathogenic mutations caused by solar UV radiation. However, an overabundance of melanin in the skin can result in photochemical damage, chloasma, and negative pigmentation (Unver et al., 2006).

TYR plays a key role in the biosynthesis of various biological melanins, including those in mammals, which involves the regulation of MITF, which is responsible for the pigment cell-specific transcription of melanin-producing enzyme genes (Oh et al., 2021). The color of human skin primarily depends on the melanin present in the skin. The expression of the TYR gene is the cause of inducing melanin biosynthesis. The increase of TYR will lead to the hydroxylation of tyrosine to 3,4-dihydroxyphenylalanine (DOPA) and the oxidation of DOPA to dopaquinone, resulting in melanin accumulation (Kameyama et al., 1993; Maranduca et al., 2019; Wasmeier et al., 2008). Melanogenesis synthesizes melanin through a series of reactions involving TYR and other melanin-related proteins (Cordero and Casadevall, 2020). Although many enzyme catalyses and chemical reactions are involved in the melanin production process, tyrosine plays a major role in melanin synthesis. Therefore, inhibition of TYR activity is the most prominent method for the developing of melanin production inhibitors. Currently, research has developed drugs such as kojic acid, arbutin, and ascorbic acid for the treatment of hyperpigmentation, they are limited by their low stability or side effects (such as skin irritation and dermatitis) (Chiari et al., 2011; Feng et al., 2022). Currently, natural extracts are becoming increasingly popular as active ingredients in cosmetics since they possess properties such as UV protection, antioxidant, antibacterial, anti-inflammatory, and anti-aging effects, with fewer side effects.

Whitening cosmetics are a primary category of skin care products and are extremely popular, and serving as a primary focus for cosmetic developers. With the rapid development of the cosmetics industry, consumers are increasingly inclined to pursue environmentally friendly, natural, and safe active ingredients, driving research toward animal and plant-based components. Natural traditional Chinese medicine ingredients offer significant advantages over synthetic chemicals, including environmental sustainability, biodegradability, enhanced safety profiles, and proven skin care benefits. Although insects receive less attention than plants in cosmetic applications, their use in traditional Chinese medicine in China spans thousands of years, with clinical experience confirming both efficacy and safety for skincare purposes. This extensive historical foundation of traditional Chinese medicine provides valuable research directions for modern cosmetics development. *Blaps rhynchopetera* Fairmaire is a kind of medicinal insect used by ethnic minorities in Southwest China for treating tumors and inflammatory conditions (Luo et al., 2023; Yang et al., 2019). Previous studies have validated its rich ingredients and nutritional profile, including 9 essential trace elements and 16 free amino acids (Dai et al., 2020). Modern pharmacological studies have shown that it has a wide range of pharmacological effects (Zhao et al., 2019), but the research on the application of its active ingredients in the field of cosmetics is still untouched. Therefore, the application of EBR in the field of cosmetics has practical value.

To study whether *B. rhynchopetera* Fairmaire has the potential to be used in cosmetics, we used different solution extraction including water extraction and ethanol extraction, to evaluate antioxidant activity, anti-melanin deposition properties, and tyrosinase inhibition capabilities. s. The study found that the EBR has good antioxidant effects, anti-melanin deposition and significant TYR inhibition. In addition, we determined the contents of phenols and flavonoids in the EBR and determined the compounds by UHPLC-Q-TOF MS. Molecular docking and MD simulation results showed that there were compounds that inhibited TYR activity in *B. rhynchopetera* Fairmaire. This research represents the first documented evidence of the cosmetic potential of *B. rhynchopetera* Fairmaire by identifying key chemical constituents responsible for whitening effects through network pharmacology approaches. Our findings establish *B. rhynchopetera* Fairmaire as a promising bioactive ingredient for the cosmetic industry, offering new opportunities for the development of natural whitening products.

## 2 Materials and methods

### 2.1 Materials

#### 2.1.1 *Blaps rhynchopetera* Fairmaire extraction

The fresh adults of *B. rhynchopetera* Fairmaire 1886 [Tenbrionidae] were collected from Yuanmou County, Chuxiong, an autonomous prefecture, in Yunnan Province, China. Water and ethanol were used to extract 1.0 kg of adults using solvent extraction. A rotary evaporator was used to evaporate the ethanol extract, and the water extract was lyophilized using a freeze-drying machine. The details are as follows.

##### 2.1.1.1 Water extraction

The 0.5 kg of *B. rhynchopetera* Fairmaire was dried in an oven and crushed by a grinder, sieved through 50 mesh, and soaked in deionized water. The volume ratio of *B. rhynchopetera* Fairmaire powder to water was 1:4, extracted 3 times, each time soaked for 72 h, filtered, and the filtrate was combined. The filtrate was concentrated to a paste under 40–45 °C and 0.06–0.1 MPa, and the WEBR was obtained. Finally, the WEBR was freeze-dried, redissolved in DMSO, and stored at − 20 °C.

##### 2.1.1.2 Ethanol extraction

The 0.5 kg of *B. rhynchopetera* Fairmaire was dried in an oven crushed into powder by a grinder, passed through a 50-mesh sieve, and soaked in 90% ethanol. The ratio of powder volume to ethanol was 1:9. Immerse at least 3 times, each time for 72 h, filter and combine the filtrate, at 40 °C–45 °C, 0.06–0.1 MPa decompression, concentrated to the paste, the relative density of 1.05–1.30, the EEBR was freeze-dried and was redissolved in DMSO and stored at − 20 °C.

### 2.2 Determination of TYR-inhibiting activity

The main reason for melanin production is that *L*-tyrosine is hydroxylated to *L*-dihydroxyphenylalanine (*L*-DOPA) under the catalysis of TYR, and then the o-diphenol is oxidized to the corresponding *L*-dopaquinone (Hearing, 1993; Prota, 1992). The inhibition of TYR was assessed using the modified dopachrome technique, with *L*-tyrosine as the substrate (Hapsari et al., 2012; Kamkaen et al., 2007). The EBR (including WEBR and EEBR) dissolved in DMSO was prepared into an initial solution with a concentration of 2 mg/mL by PBS solution, and then the initial solution was double diluted to obtain the extract solution with a concentration of 62.5 μg/mL, 125.0 μg/mL, 250.0 μg/mL, 500.0 μg/mL, 1000.0 μg/mL and 2000.0 μg/mL, respectively, as the sample to be tested. The concentration of the positive control arbutin was 2000 μg/mL.

Four test tubes A, B, C and D were taken and 250 μL of 1 mg/mL *L*-tyrosine solution was added, respectively. An additional 250 μL PBS was added to tubes A and C. Add 250 μL of the sample to be tested in tubes B and D, respectively. The solution in the four test tubes was mixed and heated in a water bath at 37 °C for 10 min. After that, 250 μL of 0.07 mg/mL TYR was added to the C and D tubes, and the same volume of PBS solution was added to the A and B tubes to fill the test sample volume. The four test tubes were placed in a water bath at 37 °C for 20 min. The above test solution was prepared by combining the contents of four test tubes and then added to a quartz cuvette. An ultraviolet spectrophotometer was used to detect the absorbance at 475 nm. The TYR inhibition rate was calculated using the following formula, with arbutin serving as a positive control. TYR inhibition rate: I = [(C-A) - (D-B)]/(C-A) × 100%.

### 2.3 The cytotoxicity in B16F10 cells

Mouse melanoma B16F10 cells were purchased from Kunming Institute Zoology, Chinese Academy of Sciences and cultured in DMEM/F12 (Gibco, Billings, MT, United States) medium supplemented with 10% fetal bovine serum (FBS) and antibiotics (100 μg/mL penicillin, 100 μg/mL streptomycin and 100 μg/mL amphotericin B), and incubated for 5% CO_2_ at 37 °C. CCK-8 cytotoxicity kit (MCE, United States) was used to detect cell viability after treatment with WEBR or EEBR or positive drug arbutin. In short, 1 × 10^6^ CFU/mL B16F10 cells were inoculated into each well of a 96-well plate. After 24 h, cells were exposed to 1 μM α-MSH and EBR at varying concentrations (1.5–200 μg/mL) for an additional 48 h and the concentration of the positive control arbutin was 200 μg/mL α-MSH is a polypeptide composed of 13 amino acids. It primarily acts on melanocytes, stimulating the conversion of tyrosine in these cells into melanin, and promoting melanocyte proliferation and melanin synthesis (Goenka et al., 2019). The blank control group comprised cells without α-MSH and EBR, while the α-MSH group was composed of cells treated with α-MSH alone. After the incubation period, each well was treated with 10 μL of CCK-8 solution and incubated for 4 h. The absorbance at 450 nm was measured using a microplate reader, and the control group was 100%. The experiment was repeated six times, and the results were expressed as a percentage change relative to the control.

### 2.4 Melanin contents and TYR-inhibiting activity determination in B16F10 cells

B16F10 cells were incubated with 1 μM α-MSH and different concentrations of EBR (1.5–200 μg/mL) for 48 h. The control group did not receive specific treatment, while the cells treated with α-MSH only constituted the α-MSH group. Arbutin was used as a positive control in the experiment and the concentration was 200 μg/mL. After washing twice with phosphate-buffered saline (PBS), the cells were lysed on ice with 1% triton X-100 solution for 10 min. Subsequently, these samples were centrifuged at 13,000 g for 10 min. The supernatant was used to measure intracellular TYR activity and the precipitate was used to quantify the melanin content of B16F10 cells (Tedasen et al., 2024). The precipitate was dissolved in 100 μL 1 mol/L NaOH at 80 °C for 30 min, and the absorbance was measured at 475 nm using a microplate reader. The absorbance of the control group was 100%. The results were reported as a percentage change relative to the control, and the experiment was set to three replicates. Subsequently, the protein concentration in the supernatant was quantified using a BCA protein quantification kit (Biosharp, Hefei, Anhui, China). In the steps, 50 μL of supernatant and 50 μL of *L*-DOPA (5 mM) were incubated at 37 °C for 1 h to measure the TYR inhibition activity, and then the absorbance was measured at 475 nm.

### 2.5 Quantitative PCR (qPCR) analysis in B10F10 cells

B16F10 cells were treated with 1 μM α-MSH and different concentrations of EBR (1.5–200 μg/mL) and 200 μg/mL of arbutin for 48 h. After incubation, the cells were washed twice with PBS and then harvested. Total RNA was isolated using a Trizol reagent. The purity and concentration of the resulting RNA were evaluated using a Nanodrop spectrophotometer. A total of 1 μg of extracted total RNA was converted into cDNA using the 5X All-In-One MasterMix Reverse Transcription Kit (ABM, United States). Subsequently, the samples were amplified using the BlasTaqTM 2X qPCR MasterMix premix kit (ABM, United States). Gene expression analysis was performed using a LightCycler^®^ 96 SW 1.1 real-time PCR system (Roche Diagnostics, Indianapolis, IN, United States). All reactions were repeated three times. The relative fold expression levels of TYR and MITF were calculated. The threshold cycle (Cq) was determined and normalized to the average level of the housekeeping gene (GAPDH) level. The relative expression level of the gene was expressed as the calculation formula ‘2^−ΔΔCt^’ and normalized by GAPDH (Livak and Schmittgen, 2001). Primers for B16F10 are shown in Supplementary Table S1. Otherwise, the dissected skin tissue was placed in the Trizol reagent to isolate RNA. The guinea pig primers involved in this experiment refer to Supplementary Table S1.

### 2.6 Western blot of cell proteins

B16F10 cells were treated with 1 μM α-MSH and different concentrations of EBR (1.5–200 μg/mL) and 200 μg/mL of arbutin. After 48 h, the cells were lysed on ice for 30 min using RIPA lysis (Sigma-Aldrich, Merck, United States) buffer, and 0.1% protease inhibitor (MedChemExpress, United States) and 0.1% phosphatase inhibitor (MedChemExpress, United States) were added to it. The proteins in the supernatant were collected after centrifugation at 13,000 × g for 15 min. A BCA protein quantification kit was used for protein determination. Each quantitative protein (30 μg) was loaded into each well of the 4%–20% SDS-PAGE gel. After SDS gel electrophoresis, the protein was transferred to 0.22 μM polyvinylidene fluoride (PVDF, Millipore, Merck, Germany) membrane. Subsequently, the PVDF membrane was blocked with 5% (w/v) bovine serum albumin (BSA, BioFroxx, Germany) at room temperature for 2 h. After the blocking, the membrane was incubated with primary antibody against TYR (Bioss, Beijing, China) and MITF (Bioss, Beijing, China) (1: 1000 dilution) at 4 °C overnight. After washing with TBST buffer containing 0.1% Tween-20 (BioFoxx, Germany) for 4 times, each time for 5 min, the membrane was incubated with secondary antibody [KPL peroxidase-labeled rabbit IgG (H + L, Seracare, United States)] at room temperature for 2 h. An advanced enhanced chemiluminescence (ECL) reagent (Tanon, Shanghai, China) was used to detect the protein bands on the PVDF membrane and photographed using a chemiluminescence analysis system (Tanon5200Multi, Shanghai, China). ImageJ software was used to quantify TYR and MITF expression levels for Western blot analysis.

### 2.7 UVB irradiation-induced pigmentation in guinea pigs

Three flowered guinea pigs (5 weeks of age, Hartley) were purchased from Chengdu Dossy Experimental Animals Co., ltd (Chengdu, Sichuan, China). We used a total of 9 guinea pigs and conducted three independent experiments, with each sample consisting of three guinea pigs. In the cage, they were placed under standard experimental conditions (22 °C ± 1 °C, 55% ± 5% humidity, 12 h of light and 12 h of dark cycle) and kept on a standard diet and free available water. After a week of adaptation, we used an electric haircut to cut off the back hair of female guinea pigs. Five independent square (1 cm × 1 cm) dorsal areas of each guinea pig were exposed to UVB radiation (305 nm, SANIKYO, Japan). Guinea pigs were exposed to UVB light for 3 weeks at intervals of 1 day, 1 h per day, starting from the second day after shaving (Yoshida et al., 2007). The arbutin (positive control), EBR including WEBR and EEBR were dissolved in a mixture of ethanol and water (1: 9, v/v). From the second day after the last UVB irradiation, each sample solution was applied locally with a pipette to separate the shaving area once a day for 2 weeks. After that, the guinea pigs were anesthetized and sacrificed, and the skin tissues of different samples were taken for subsequent experiments. The use of guinea pigs in research was authorized following the ethical approval application submitted by the Kunming Institute of Zoology, Chinese Academy of Sciences (Ethical registration number: IACUC-RE-2024-08–011).

### 2.8 Melanin and TYR inhibition activity determination in animal skin tissues

The skin tissue was incubated in 2 mol/L NaBr at 37 °C for 5 h to separate the epidermis from the dermis. To measure the melanin content, the epidermis was dissolved in 250 μL of 1 mol/L NaOH containing 10% DMSO and treated at 80 °C for 1 h and centrifuged 13, 000 × g for 30 min. The absorbance of the supernatant was measured at 475 nm. For the determination of TYR activity, the isolated epidermis was placed in a 1% Triton X-100 (Beyotime, Shanghai, China) containing 0.1 mM PMSF and ground in a grinding machine. The supernatant was collected by centrifugation at 13,000 *g* for 30 min at 4 °C. Subsequently, the protein concentration in the supernatant was quantified using a BCA protein quantification kit (Biosharp, Hefei, Anhui, China). In the steps, 50 μL of supernatant and 50 μL of *L*-DOPA (5 mM) were incubated at 37 °C for 1 h to measure the TYR inhibition activity, and then the absorbance was measured at 475 nm.

### 2.9 Western blot analysis in animal skin tissues

The dissected skin tissue was placed in the RIPA lysis (Sigma-Aldrich, Merck, United States) solution of 0.1% protease inhibitor and 0.1% phosphatase inhibitor, ground for 20 min in a grinder, and centrifuged at 13,000 *g* at 4 °C for 30 min. To maintain the protein concentration, the protein concentration in the supernatant was quantified using a BCA protein quantification kit (Biosharp, Hefei, Anhui, China). The following Western blot steps are shown in Materials and Methods 2.7. ImageJ software was used to quantify TYR and MITF expression levels for Western blot analysis.

### 2.10 IHC analysis

Tissue sections were fixed with 4% paraformaldehyde, dewaxed, and dehydrated, and then washed with phosphate-buffered saline (PBS, pH = 7.2) to remove excess reagents. The sections were then incubated for 15 min in boiling citrate buffer (10 mM, pH 6.0) to restore the antigenic sites. Next, the slices were treated with 3% H_2_O_2_ for 25 min and washed three times in PBS (pH 7.4) on a horizontal shaker (Qilinbeier, Jiangsu, China) to inhibit endogenous peroxidase activity. This was followed by a 15-min blocking step with 2% BSA at 37 °C. Subsequently, the sections were incubated overnight at 4 °C with purified rabbit polyclonal antibodies diluted 1:1,000, followed by washing with PBS (pH 7.4). The sections were then exposed to a secondary antibody (KPL peroxidase-labeled rabbit IgG [H + L, Seracare, United States]) for 50 min at room temperature. The immunoperoxidase reaction was carried out using 3,3-diaminobenzidine (Servicebio, Wuhan, China), and the sections were counterstained with hematoxylin. The primary antibody was substituted in the negative control with an antigen-free antibody. Images were acquired using a slide scanning system (Teksqray, Shenzhen, China) and ImageJ software was used to quantify TYR and MITF expression levels in guinea pig skin tissues.

### 2.11 Determination of total phenol content

The 0.03 g insect extraction of water and ethanol was dissolved with 0.6 mL 60% ethanol and then ultrasound for 5 min and centrifuged at 12,000 *g* for 10 min at 25 °C. The supernatant was diluted to 0.6 mL with 60% ethanol. The 2 mg/mL tannic acid standard solution was diluted to 0.025, 0.05, 0.1, 0.2, 0.3, 0.4 mg/mL. The total phenol content of the EBR (The concentration was 0.39–25.00 μg/mL) was determined by the total phenol detection kit, according to the manufacturer’s instructions (Norminkoda, Wuhan, China) and mixed well at room temperature for 30 min, and the absorbance was read at 750 nm by ultraviolet spectrophotometer, and the formula: △A = A_determination_ - A_control_, △A’ = A_standard_ - A_blank_. The standard curve ‘y = kx + b’ was drawn with the concentration of tannic acid as abscissa and △A′ as ordinate, and △A was brought into the formula to obtain x. Total phenol content (mg/g mass) = x × V1/(W × V1/V) × D (V: Add extract volume, 0.6 mL; V1: sample volume in the reaction: 0.05 mL; W: sample quality: g; D: dilution multiple, undiluted is 1).

### 2.12 Determination of total flavonoid content

The 0.03 g insect extract was dissolved in 0.6 mL of 60% ethanol and then sonicated for 5 min, followed by centrifugation at 12,000 *g* for 10 min at 25 °C. The supernatant was diluted to 0.6 mL with 60% ethanol. The 10 mg/mL rutin standard solution was diluted to 0.005, 0.01, 0.02, 0.039, 0.078, 0.156, 0.3125, 0.625, and 1.25 mg/mL. The total flavonoid content of the EBR (The concentration was 0.39–25.00 μg/mL) was determined by the total flavonoid content detection kit, according to the manufacturer’s instructions (ZCi Bio, Shanghai, China) and mixed well at 37 °C water bath for 45 min, centrifuged at 10,000 *g* for 10 min to take the supernatant, and the absorbance was read at 510 nm by a microplate reader (Epoch Etock; Biotek, United States), and calculated using the formula: △A = A_determinationm_ - A_control_, △A’ = A_standard_ - A_blank_. The standard curve ‘y = kx + b’ was drawn with the concentration of rutin as abscissa and △A′ as ordinate, and △A was brought into the formula to obtain x. Total flavonoids content (mg/g fresh weight) = x × V/W (V: Add extract volume, 0.6 mL; W: sample quality: g).

### 2.13 Ultra-high performance liquid chromatography with quadrupole time-of-flight mass spectrometry (UHPLC-Q-TOF MS)

The appropriate amounts of samples including water extracts and ethanol extracts (0.5 mL) were added to precooled methanol/acetonitrile/water solution (2:2:1, v/v), vortex mixing, low temperature ultrasonic 30 min, −20 °C standing 10 min, 14,000 × g centrifugation for 20 min at 4 °C, the supernatant was vacuum dried, 100 μL solution (acetonitrile: water = 1:1, v/v) was added to the mass spectrometry analysis, vortex, 14,000 × g centrifugate to 15 min at 4 °C, the supernatant was injected for analysis.

#### 2.13.1 Chromatographic condition

The samples were separated by ultra-high performance liquid chromatography (UHPLC, Agilent 1290 Infinity LC, United States) and an HILIC chromatographic column (Agilent, United States) at 25 °C. The flow rate was 0.5 mL/min. The injection volume was 2 μL; mobile phase solvent A: water with 25 mM ammonium acetate and 25 mM ammonia, solvent B: acetonitrile; the gradient elution procedure was as follows: 0–0.5 min, 95% B; 0.5–7 min, B from 95% linear change to 65%; 7–8 min, B changed linearly from 65% to 40%; 8–9 min, B maintained at 40%; 9–9.1 min, B from 40% linear change to 95%, 9.1–12 min, B maintained at 95%; during the whole analysis process, the sample was placed in a 4 °C automatic sampler. To avoid the influence caused by fluctuations in the instrument detection signal, the continuous analysis of the sample was carried out in a random order, and six repetitions were performed.

#### 2.13.2 Mass spectrometer conditions

The samples were separated using ultra-high performance liquid chromatography (UHPLC, Agilent 1290 Infinity LC, United States) and a HILIC column (Agilent, United States). They were then analyzed by a mass spectrometer (AB SCIEX Triple TOF 6600, United States) in both ESI+ and ESI- modes.

The parameters of the ESI source are as follows: nebulizer-assisted heating gas 1 (Gas1): 60, assisted heating gas 2 (Gas2): 60, curtain gas (CUR): 30 psi, ion source temperature: 600 °C, spray voltage (ISVF) ± 5500 V (positive and negative modes); the first-order mass-to-charge ratio detection range: 60–1000 Da, the second-order mass-to-charge ratio detection range: 25–1000 Da, the first-order mass spectrometry scan cumulative time: 0.20 s/spectra, and the second-order mass spectrometry scan cumulative time: 0.05 s/spectra. The second-order mass spectrometry was obtained using a data-dependent acquisition mode (IDA), and the peak intensity screening mode was employed. The cluster voltage (DP): ±60 V (positive and negative modes), collision energy: 35 ± 15 eV, and IDA is set as follows: dynamic exclusion of isotope ion range: 4 Da. Each scan collected 10 fragments. The supernatant of each test sample is resuspended in 10 μL and used as a quality control (QC) sample. A QC sample is introduced for every six samples to evaluate the stability of the equipment. Six replicates were set in the experiment.

#### 2.13.3 Data analysis

The original data was converted into mzXML format by ProteoWizard, and XCMS software was used for peak alignment, retention correction and peak area extraction. Metabolite structure identification and data preprocessing were performed on the data extracted by XCMS, and then quality evaluation and data analysis were performed on the experimental data.

### 2.14 Network pharmacology

The phenols and flavonoids in *B. rhynchopetera* Fairmaire were imported into the PubChem database (https://pubchem.ncbi.nlm.nih.gov/) to obtain their SMILES structures. They were used to predict targets on the SwissTargetPrediction platform (http://www.swisstargetprediction.ch/), and targets with scores greater than 0.2 were selected. Potential targets related to melanin deposition were retrieved from the GeneCards database (https://www.genecards.org) (correlation score >3), OMMI database (https://www.omim.org), and DisGeNET database (https://www.disgenet.org) with ‘Melanin deposition’, ‘TYR inhibition’ as the keywords (Xu J. et al., 2024). The intersection of disease targets and component targets was taken as the potential effect of EBR on improving melanin deposition, and the Venn diagram was drawn by an online mapping website (https://www.bioinformatics.com.cn) (Tang et al., 2023).

Through the above process, we identified the targets of active ingredients related to melanin deposition and TYR inhibition from *B. rhynchopetera* Fairmaire*.* To illustrate the relationship between the primary protein targets of the drug, a protein-protein interaction (PPI) network was generated using the STRING website (https://cn.string-db.org) and visualized using Cytoscape 3.10.1 software. In addition, a complex target-pathway-network was created using Cytoscape 3.10.1 software. Compounds, proteins, and signal pathways are represented as nodes in the graph network, while the interactions between compounds, proteins, and signal pathways are shown as edges. The nodes in the graphical network represent many substances, targets, and related diseases. The edges between them show the connection between the composite target and the target disease. After analyzing the values of ‘degree’, ‘closeness’, and ‘betweenness’, the target with a comprehensive score greater than the average of 0.900 and a degree greater than the average is selected as the key target.

### 2.15 GO and KEGG analysis

DAVID database (https://david.ncifcrf.gov) was used to perform Gene Ontology (GO) and Kyoto Encyclopedia of Genes and Genomes (KEGG) enrichment analysis on the potential targets of the EBR to improve melanin deposition and inhibit TYR activity. Enrichment analysis was performed to predict the main targets and possible biological activity mechanisms of the active components of the EBR to improve melanin deposition and play a whitening role. GO enrichment includes molecular function (MF), biological process (BP) and cellular component (CC) and these analyses are carried out on the Wei Sheng Xin platform (https://www.bioinformatics.com.cn).

### 2.16 Molecular docking

According to the results of KEGG pathway enrichment, the pathways most closely related to melanin deposition and the TYR metabolism mechanism were identified, and the corresponding active components were molecularly docked. The top four core compounds were selected as therapeutic small molecules. The SDF format of the needed compounds was retrieved from the PubChem database and converted to the MOL format using PyMOL software. To determine the 3D structure of the protein, the SWISS-MODEL database (https://swissmodel.expasy.org) was used to retrieve the PDB format export, and then PyMOL software was employed to remove excess chains, including ligands, dehydrated and hydrogenated proteins, and drug molecules, and to set the docking box. Semi-flexible docking was performed on the identified molecules using Mgltools software (version 1.5.6) and AutoDock Vina software (Eberhardt et al., 2021; Trott and Olson, 2010) to find the binding energy and position of each component of *B. rhynchopetera* Fairmaire. The docking state with a binding energy of <−7.0 kcal/mol was considered to be good (Zhu et al., 2024), and the molecular docking results were visualized using PyMOL software.

### 2.17 MD simulation

By comparing the binding energies of quercetin, luteolin, myricetin, apigenin, and TYR, as well as AKT1 and EGFR using molecular docking, we selected TYR and the aforementioned four compounds for MD simulation. To better understand the complex interaction processes between the TYR protein and the four candidate small molecules, this study employs GROMACS software (version 2024.2) to predict the dynamic changes between the biomacromolecule and the ligands (Lemkul, 2018). To ensure the MD simulation runs correctly, the protein and ligands must be converted into GROMACS-compatible force field files for the simulation. The OPLS-AA/L all-atom force field was selected, and the TIP3P model was used as the solvent. The boundaries of the simulation box were set to 1 nm × 1 nm × 1 nm. In the simulation box, one molecule of TYR protein and one molecule of the ligand were included, while the remaining space was filled with water molecules (spc216). Subsequently, sodium and chloride ions were added to neutralize the system’s charge, and energy minimization was performed to optimize the system components.

In the initial state of the protein-ligand complex, there might be excessively short distances between atoms, conflicts, or unnatural interactions. If the dynamics simulation is started directly, these unreasonable conditions may destabilize the system or even cause the simulation to fail. Through energy minimization and equilibration steps, these issues can be mitigated, allowing the system to relax under mild conditions and avoid drastic structural changes gradually. Thus, hydrogen atoms in the ligand must be constrained before proceeding with the NVT (constant Number of particles, Volume, and Temperature) and NPT (constant Number of particles, Pressure, and Temperature) calculations. The MD simulation was executed under the NPT ensemble for a duration of 50 nanoseconds at 300 K (using C-rescale for temperature control) and 1.0 bar (Childers and Daggett, 2018). The system maintained constant volume and used the Smooth Particle Mesh Ewald (PME) method for efficient long-range electrostatic interactions.

Finally, MD simulations were performed to obtain curves representing protein-ligand interactions, such as the RMSD and RMSF of the protein and ligand. For analyzing the dynamics of the protein-ligand interactions, interaction energy calculations should not be considered part of a regular simulation (Tedasen et al., 2024). Therefore, the average short-range Coulombic interaction energy and the short-range Lennard-Jones energy were used. The total interaction energy, however, is useful in this case.

### 2.18 Statistical analysis

All performed experiments were undertaken in triplicate (n = 3) and the results were conducted utilizing GraphPad Prism 6.01 (GraphPad Software, San Diego, CA, United States). Statistical differences were assessed using a one-way ANOVA. The data are presented as mean *±* standard deviation (mean *± SD*), with statistical significance defined as *p < 0.05, **p < 0.01.

## 3 Results

### 3.1 EBR has TYR inhibition activity

**FIGURE 1:**
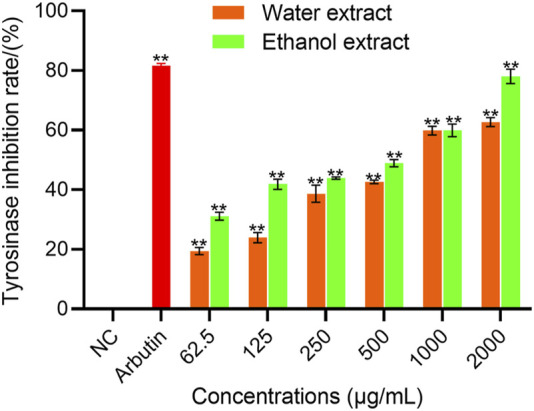
TYR inhibition activity of WEBR and EEBR. Data represent mean ± *SD* of three independent experiments. NC was referred to as blank control. One-way ANOVA was performed, **p < 0.01, *p < 0.05, compared with NC group.

The potential of EBR as a whitening agent to inhibit melanin formation was evaluated by measuring TYR inhibitory activity. As shown in Figure 1, compared with the blank control, there were significant differences in different concentrations of WEBR and EEBR (*p* < 0.01). With the increase in different concentrations, the inhibition rate of WEBR and EEBR on TYR activity increased gradually. When the concentration was 1000.0 μg/mL, the inhibition rate of WEBR and EEBR on TYR activity could reach more than 50%.

### 3.2 The cytotoxicity, melanin-inhibiting activity and TYR-inhibiting activity of EBR in B16F10 cells

**FIGURE 2:**
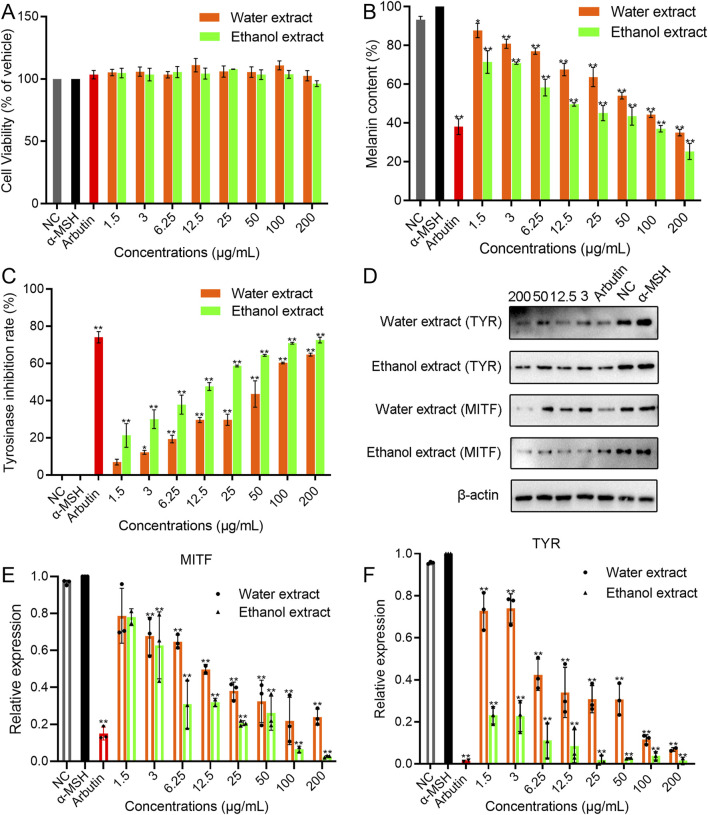
The cytotoxicity, melanin-inhibiting activity, and TYR-inhibiting activity of EBR in B16F10 cells. **(A)** Cytotoxicity of EBR. **(B)** Melanin-inhibiting activity for EBR. **(C)** TYR-inhibiting activity for EBR. **(D)** Western blotting analysis for EBR. **(E)** qPCR analysis of the TYR gene for EBR. **(F)** qPCR analysis of the MITF gene for EBR. Data represent mean ± *SD* of three independent experiments. NC was referred to as a blank control. One-way ANOVA was performed; **p < 0.01, *p < 0.05, compared with α-MSH group.

As shown in Figure 2A, compared to the blank control (PBS), the cell viability of WEBR and EEBR was above 90% in the range of 1.5–200 μg/mL. When the concentration reached 100 μg/mL, the cell viability reached 100%, indicating that the EBR should be safe and reliable in the field of cosmetics.

As shown in Figure 2B, as expected, α-MSH treatment significantly increased the melanin content in B16F10 cells. Compared with the α-MSH group, the concentration of EBR at 100 and 200 μg/mL exhibited a significant inhibitory effect on the melanin synthesis of B16F10 cells. The content of melanin eliminated by 200 μg/mL WEBR was 35.040% ± 1.683%, and the content of melanin eliminated by 200 μg/mL EEBR was 25.325% ± 4.094%, respectively, showing a certain concentration dependence. Furthermore, different concentrations of EBR treatment had a significant inhibitory effect on TYR activity in B16F10 cells (Figure 2C). Under a concentration range of 1.5–200.0 μg/mL, the inhibitory effect gradually increased with increasing concentration, exhibiting a concentration-dependent effect.

Melanin production is regulated by enzyme cascades including TYR. The expression of TYR is regulated by the transcription factor MITF (Liu and Fisher, 2010). Western blot analysis showed that the protein expression levels of TYR and MITF were significantly downregulated after α-MSH treatment (Figure 2D; Supplementary Figure S1). It indicated that the EBR significantly inhibited the level of TYR and MITF protein.

qPCR was used to detect the expression of TYR and MITF mRNA in B16F10 cells induced by the EBR. The expression of β-actin mRNA remained stable under varying concentrations of EBR, confirming that no other factors interfered with the expression of TYR and MITF mRNA during the experiment (Figures 2E,F). Compared with α-MSH, the expression of TYR and MITF mRNA was significantly inhibited by different concentrations of EBR for 48 h, especially with EEBR. The expression of TYR and MITF mRNA decreased with increasing EBR concentration. It is speculated that the EBR inhibits the production of melanin by inhibiting the activity of TYR and MITF gene.

### 3.3 The melanin-inhibiting activity and TYR-inhibiting activity of EBR in guinea pigs

**FIGURE 3:**
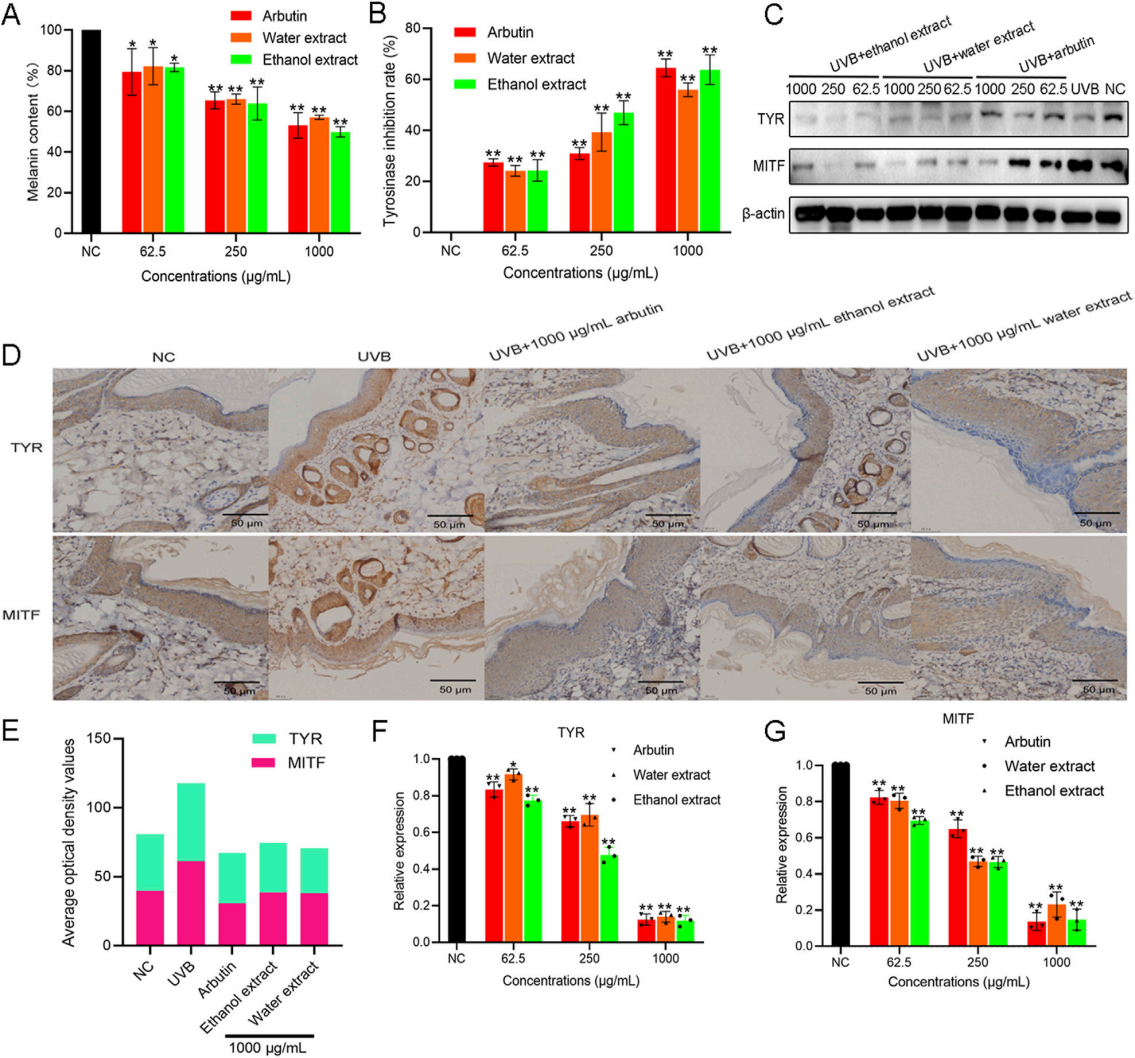
The melanin-inhibiting activity and TYR-inhibiting activity of EBR in guinea pigs. **(A)** Melanin-inhibiting activity for EBR. **(B)** TYR-inhibiting activity for EBR. **(C)** Western blot analysis for EBR. **(D)** Expression of TYR and MITF in the skin of guinea pigs was observed by IHC. **(E)** ImageJ analysis of TYR and MITF expression levels in the skin of guinea pigs for EBR. **(F)** qPCR analysis of the TYR gene for EBR. **(G)** qPCR analysis of the MITF gene for EBR. Data represent mean ± *SD* of three independent experiments. NC was referred to as a blank control. One-way ANOVA was performed; **p < 0.01, *p < 0.05, compared with the NC group.

The effect of EBR on melanin deposition in guinea pig skin was detected by measuring melanin content and TYR activity. The melanin content of the control treated with UVB alone was 100%. The results showed that the deposition of melanin decreased with an increase in the concentration of the EBR, indicating a concentration-dependent effect (Figure 3A). Treated with arbutin and the EBR, the melanin content of arbutin was 53.120% ± 6.237%, the content of WEBR was 57.070% ± 1.047%, and the content of EEBR was 49.895% ± 2.539%. Compared with the control group, the effect of EEBR on eliminating melanin deposition in guinea pig skin was slightly stronger than that of arbutin, indicating that the EBR eliminated melanin deposition.

The TYR activity treated with only UVB was assigned to 100%. The results showed that the inhibitory activity of TYR increased with the increase in the concentration of EBR, exhibiting a concentration-dependent effect (Figure 3B). When the concentration of the EBR was 1000 μg/mL, the inhibition rate of TYR by the WEBR was 55.893% ± 2.672%, and the inhibition rate of TYR by the EEBR was 63.780% ± 5.792%. At the same time, the inhibition rate of TYR by arbutin at 1000 μg/mL was 64.483% ± 3.485%. The TYR inhibition rate of EEBR was equivalent to that of arbutin.

To elucidate the inhibitory effect of EBR on melanin synthesis, we determined the expression of TYR and MITF proteins by Western blot analysis (Figure 3C) and mRNA levels by qPCR (Figures 3F,G). Compared with UVB treatment, arbutin and EBR inhibited the expression of TYR and MITF in guinea pig epidermal cells. The expression of TYR and MITF mRNA decreased with increasing EBR concentration. The inhibition of protein expression is consistent with the inhibition of gene expression (Figure 3C; Supplementary Figure S1). It is speculated that the EBR inhibits melanin production by suppressing the expression of TYR and MITF genes, thereby reducing the protein levels of TYR and MITF in the epidermis of guinea pigs, which in turn inhibits cell melanin production.

To evaluate the content of melanin in the epidermis of guinea pigs treated with EBR, the results of immunohistochemical antibody identification showed that compared with the samples treated with UVB irradiation alone, the positive expression of epidermis treated with arbutin and EBR including WEBR and EEBR decreased (the positive expression of DAB was brownish yellow) (Figures 3D,E). The results showed that the activity of TYR and MITF protein was decreased, which could further indicate that the EBR can eliminate the increase of melanin content stimulated by UVB.

### 3.4 EBR are rich in phenols and flavonoids

**FIGURE 4:**
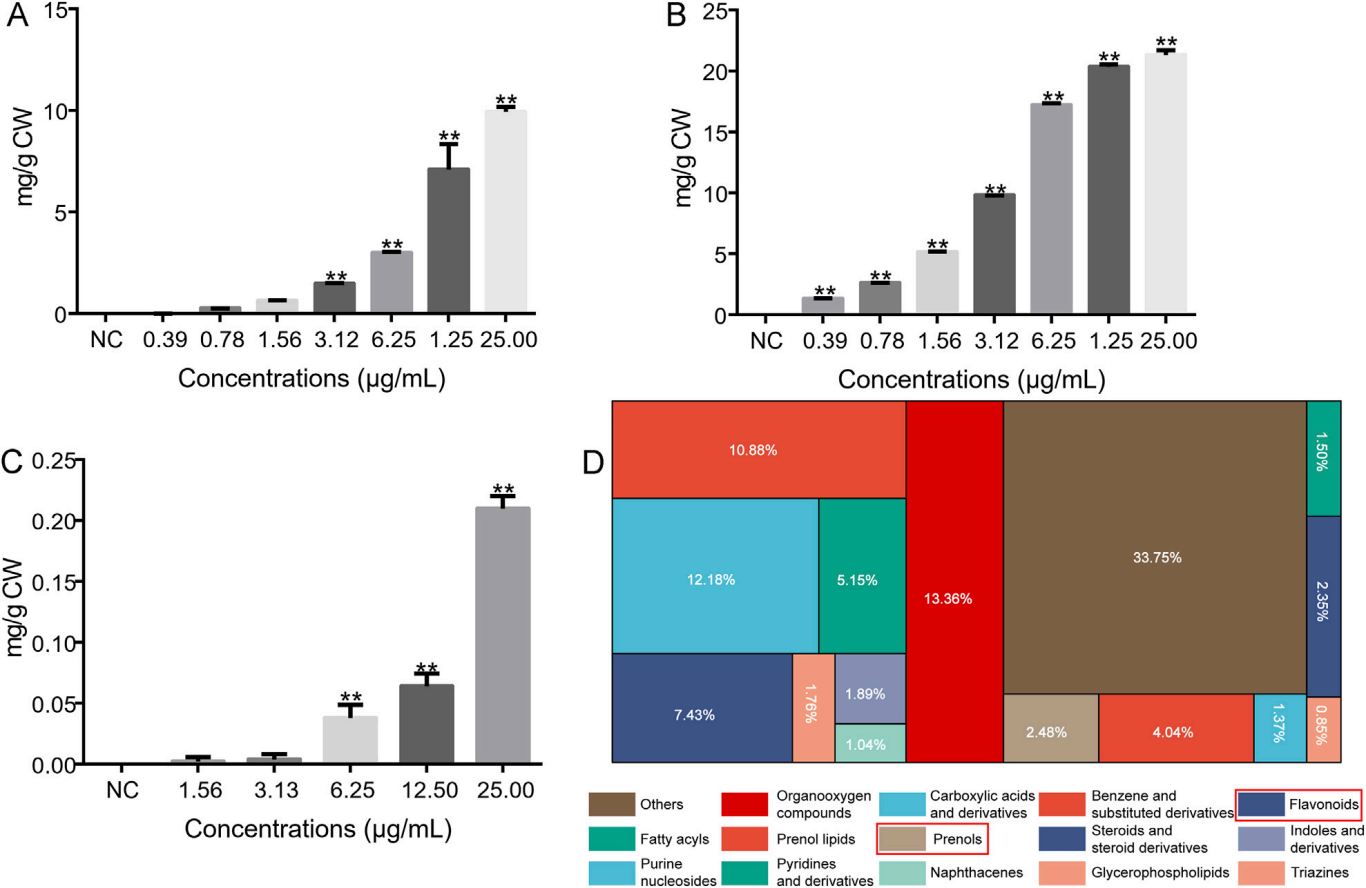
Total flavonoids and phenolics in EBR. **(A)** Total phenol content of WEBR. **(B)** Total phenol content of EEBR. **(C)** Total flavonoid content of EEBR. **(D)** The proportion of identified metabolites in each chemical classification. NC was referred to as blank control. One-way ANOVA was performed; **p < 0.01, *p < 0.05.

In this study, the contents of total phenols and total flavonoids were characterized. Tannic acid was used as a standard to determine the total phenol content. The linear equation of standard tannic acid was y = 2.5204x + 0.0253; *R* ^2^ = 0.9976 (Supplementary Figure S3A). Using the standard tannic acid calibration curve equation, the total phenol content obtained from the WEBR and EEBR is shown in Figures 4A,B. The total phenol content of EEBR was higher than that of WEBR, and with the increase of concentration, the total phenol content of EEBR and WEBR increased gradually, indicating that both EEBR and WEBR contained phenolic compounds. Rutin was used as a standard to determine the content of total flavonoids. The standard linear equation was y = x + 1E-16, *R* ^2^ = 1 (Supplementary Figure S3B). As shown in Figure 4C, with the increase of EEBR concentration, the flavonoid content gradually increased, while the WEBR did not detect any total flavonoid content, which may be attributed to the insolubility of flavonoids in water, resulting in the inability to detect the presence of flavonoids in the WEBR.

To determine the total phenols and total flavonoids in the EBR (WEBR and EEBR), we carried out UHPLC-Q-TOF MS analysis. The results showed that the response intensity and retention time of each chromatographic peak overlapped, indicating that the variation caused by instrument error was minimal throughout the experiment, which ensured the repeatability and reliability of the data. The main peaks observed in the chromatogram were attributed to positive and negative modes using ESI-MS (Supplementary Figure S4). The OPLS-DA analysis was performed on the peaks extracted from the WEBR and the EEBR to examine the concentration and distribution of metabolites in the groups and the differences between them. The results showed that samples from different groups (WEBR and EEBR) could be distinguished, while samples from the same group had a more concentrated metabolite distribution, indicating that the collected data could be used to identify different compounds between groups (Supplementary Figure S5A, 5B. After the combination of positive and negative ions, a total of 1535 compounds were identified, including 1003 positive ion compounds and 535 negative ion compounds.

Phenols and flavonoids are recognized as natural secondary metabolites that have been demonstrated to possess potential benefits, including antioxidant, antibacterial, and anti-inflammatory effects. Many phenols and flavonoids have been reported to inhibit TYR activity, which in turn inhibits melanin production (Gillbro and Olsson, 2011; Zhu and Gao, 2008), and have anti-aging properties due to their antioxidant and anti-inflammatory properties (Ferreira et al., 2021; Silva et al., 2017). The biological activity of the extract is closely related to the content of total flavonoids and phenols. Therefore, in this study, UHPLC-Q-TOF MS was used to characterize the possible compounds in the EBR. The results showed that there were 1538 compounds in the EBR, including phenols, flavonoids, carboxylic acids and derivatives, benzene and substituted derivatives, organooxygen compounds, organooxygen compounds, steroids, and steroid derivatives, etc (Figure 4D). Total phenols accounted for 2.48%, and flavonoids accounted for 7.43%. Among them, 54 kinds of flavonoids and 28 kinds of phenolic compounds were identified (Supplementary Table S2).

### 3.5 Discovering whitening compounds through network pharmacology

**FIGURE 5:**
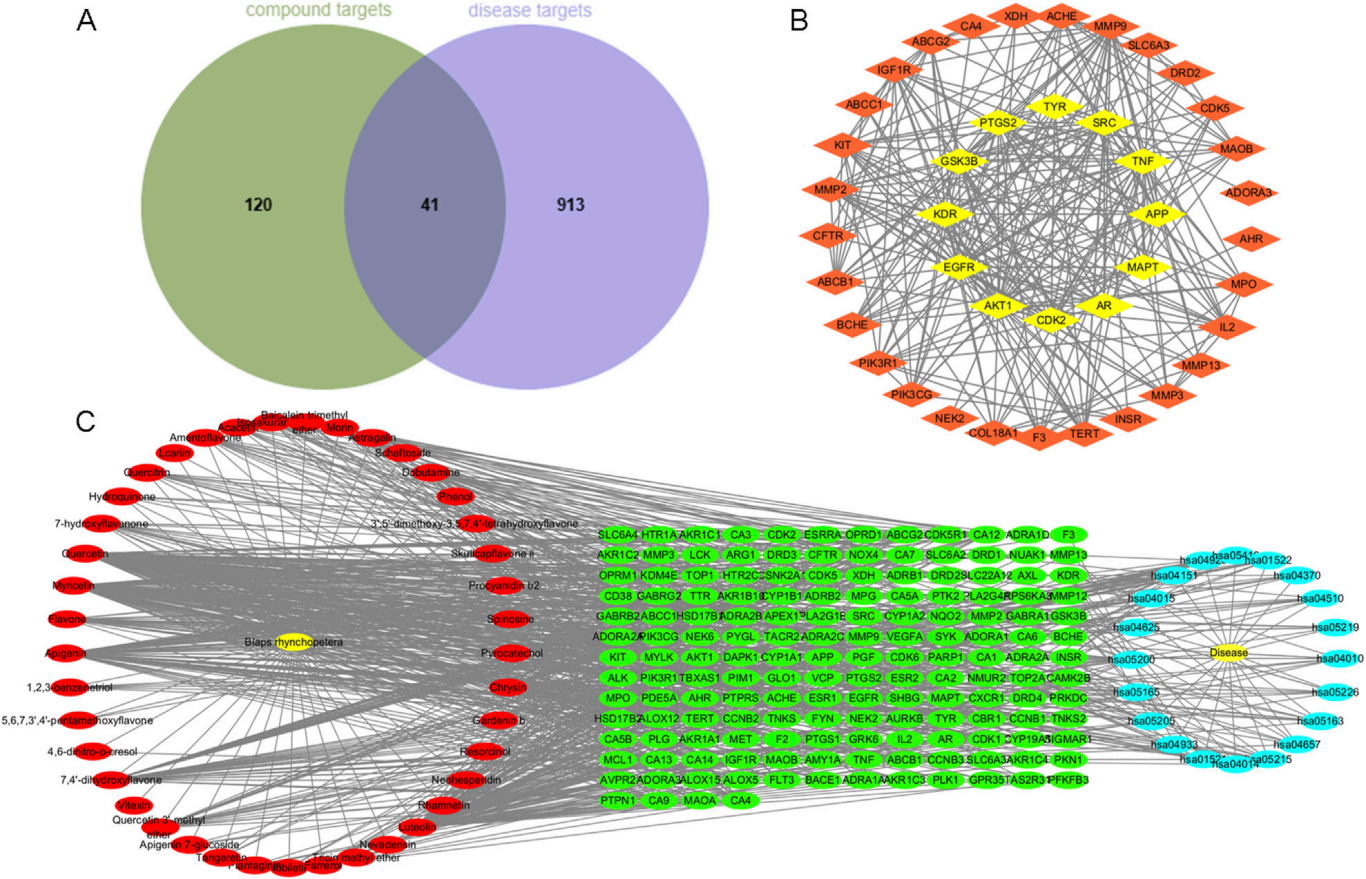
Diagram of component targets and disease targets and “Component-Target-Disease” network. **(A)** Venn diagram of component targets and disease targets of *Blaps rhynchopetera* Fairmaire. **(B)** The Cytoscape plugin Centiscape2.2 highlights the top 12 hub genes in the PPI network, where their importance is underscored by node coloration, with darker and yellower hues signifying greater scores. **(C)** “Component-Target-Disease” network of *Blaps rhynchopetera* Fairmaire.

The Venn diagram of 161 compound targets and 954 disease targets was drawn. The intersection part was a total of 41 targets of phenols and flavonoids of EBR acting on melanin and TYR (Figure 5A). These intersection targets were imported into the string database and screened the value of ‘degree’, ‘closeness’, and ‘betweenness’ to obtain the interaction diagram between the target and the target, as shown in Figure 5B and Supplementary Figure S6. Based on Cytoscape 3.10.0 software, a compound-target-disease network was constructed to predict the relationships between the active components, targets, and diseases of *Blaps rhynchopetera* Fairmaire, and the key compounds inhibiting melanin deposition and TYR inhibition were identified (Figure 5C). The network consists of 225 nodes and 933 edges, with an average degree of 8.29. Each edge represents the interaction between chemical components and targets, and the higher the degree, the more critical its role in the interaction network. According to the compound-target-disease network, quercetin had the highest number of docking targets (72), followed by myricetin (56), luteolin (34), apigenin (32), etc. Among the 161 targets, EGFR had the most docking compounds (21), followed by AKT1 (19) and TYR (14), indicating that these components and targets may play a crucial role in the process by which *B. rhynchopetera* Fairmaire exerts its skin whitening effect.

### 3.6 Signal pathway enrichment analysis reflects the whitening mechanism of *Blaps rhynchopetera* Fairmaire

**FIGURE 6:**
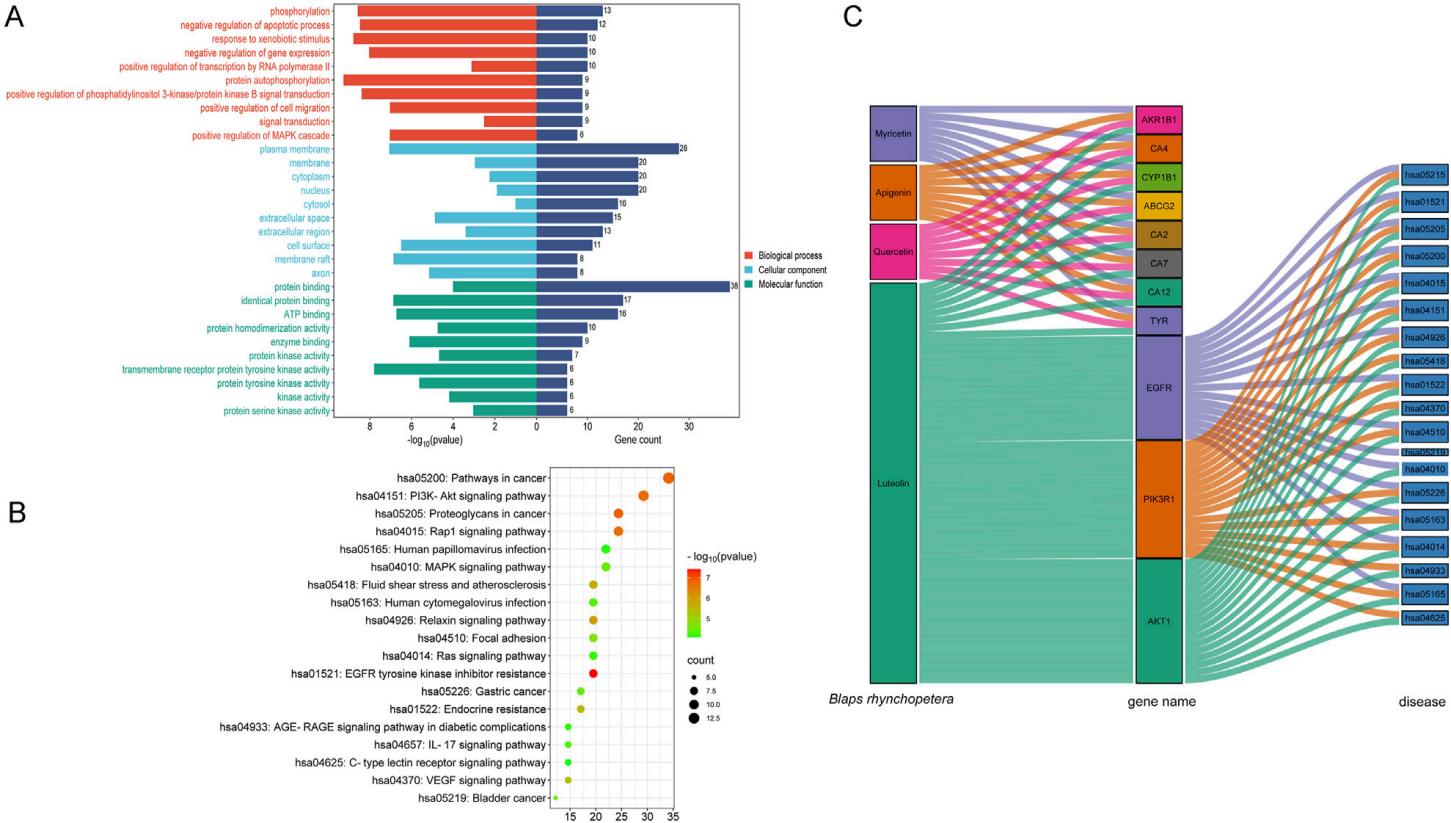
Go enrichment, KEGG enrichment, and network diagram (after screening) of *Blaps rhynchopetera* Fairmaire in treating melanin deposition and TYR inhibition. **(A)** Enrichment of gene ontology (GO) categories in potential intersection targets of *Blaps rhynchopetera* Fairmaire related to melanin deposition and TYR inhibition. The bubble plot of the top 10 GO categories in the three domains of biological process (BP), cellular component (CC), and molecular function (MF). **(B)** KEGG enrichment analysis of the top 20 signaling pathways for anti-melanin deposition and TYR inhibition of *Blaps rhynchopetera* Fairmaire. **(C)** ‘Component-Target-Disease’ network diagram of *Blaps rhynchopetera* Fairmaire in treating melanin deposition (after screening).

Based on the DAVID database, GO and KEGG enrichment analysis was performed on 41 potential melanin deposition and TYR inhibition targets of *Blaps rhynchopetera* Fairmaire, and 260 items of biological process (BP) (*p* < 0.05), 44 items (*p* < 0.05) cellular component (CC), and 72 items (*p* < 0.05) of molecular function (MF) were obtained. According to the number of genes, the top 10 were selected for GO function analysis and the top 20 were selected for KEGG enrichment analysis (Figures 6A,B). GO function analysis indicated that the biological processes mainly involve phosphorylation, negative regulation of the apoptotic process, response to xenobiotic stimulus, and negative regulation of gene expression, among others, which occur primarily in the plasma membrane, membrane, cytoplasm, nucleus, and other cellular compartments (Figure 6A). KEGG enrichment showed that pathways in cancer, PI3K-Akt signaling pathway, MAPK signaling pathway, *etc.*, Were ranked at the top, suggesting that the *B. rhynchopetera* Fairmaire may exert antioxidant, anti-melanin, and anti-TYR through these pathways (Figure 6B). Based on KEGG enrichment analysis and the ‘Component-Target-Disease’ network of *B. rhynchopetera* Fairmaire in Figure 5C, we further screened the top four key components (degree value >55) and the top 11 key targets (degree value >20) according to the degree value, and constructed the ‘Component-Target-Disease’ interaction network of *B. rhynchopetera* Fairmaire (Figure 6C).

### 3.7 Molecular docking of targets-compounds

**FIGURE 7:**
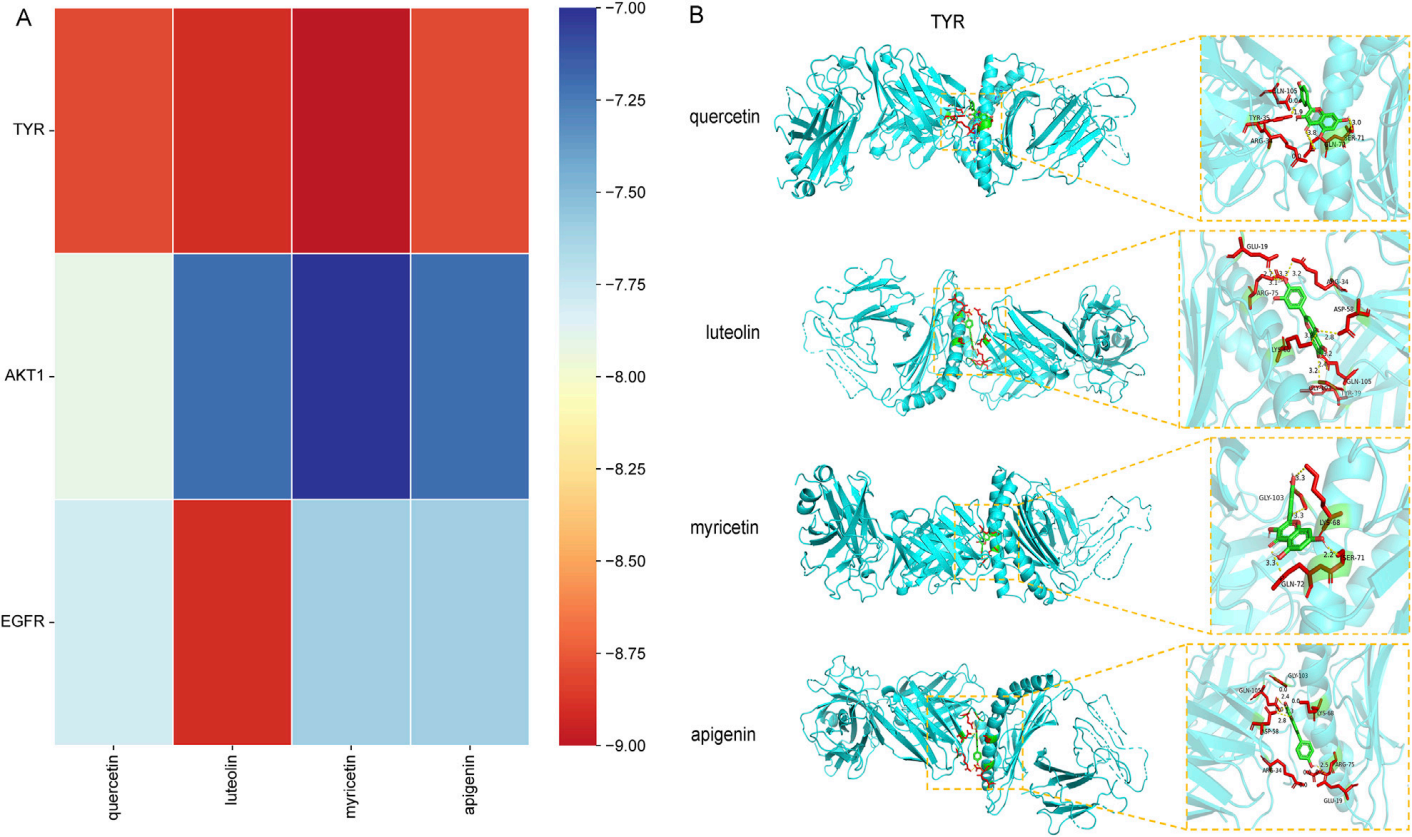
Component-target molecular docking score heat map and visual analysis of molecular docking. **(A)** Component-target molecular docking score heat map of *Blaps rhynchopetera* Fairmaire in the treatment of anti-TYR inhibitory activity and anti-melanin deposition. **(B)** Visual analysis of molecular docking for quercetin-TYR, luteolin-TYR, myricetin-TYR, and apigenin-TYR.

To verify the reliability of the screened compound-target interaction, molecular docking analysis selected the top three proteins. According to the results of the ‘Component-Target-Disease’ interaction network analysis from Figure 5C, the targets TYR, Akt1 and EGFR with higher degree values were selected for molecular docking simulation with the main active components quercetin, myricetin, luteolin and apigenin of *Blaps rhynchopetera* Fairmaire. The results of molecular docking show that the binding activity between the receptor and the ligand is expressed by the binding energy, and the binding energy <0 kJ/mol can be used to determine the spontaneous binding ability of the ligand and the receptor. The smaller the binding energy, the higher the affinity of the receptor and ligand, the greater the possibility of action. The binding energy <−5.0 kJ/mol indicates that the binding activity is high, and the binding energy <−7.0 kJ/mol indicates that the binding activity is strong. The results showed that the binding energies of active components including quercetin, myricetin, luteolin and apigenin in *B. rhynchopetera* Fairmaire to target protein TYR, AKT1, and EGFR were all <−7.0 kJ/mol, indicating that the binding ability was very high, as shown in Figure 7A and visualized by PyMOL software which shown in Figure 7B.

### 3.8 MD simulation of targets-compounds

**FIGURE 8:**
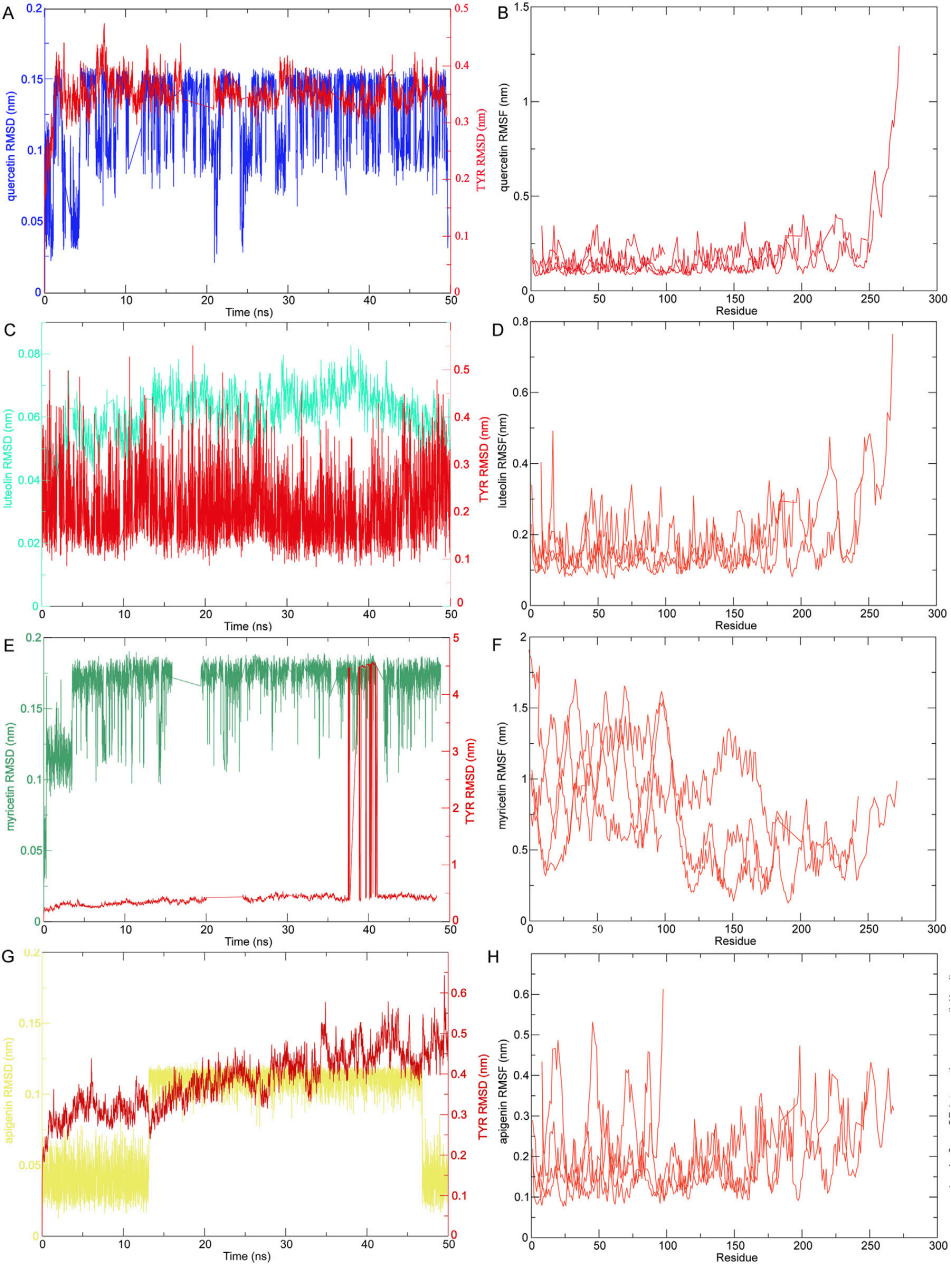
The RMSD and RMSF of MD simulation of compounds-targets. **(A)** MD simulation ligand-protein interaction RMSD profile of quercetin-TYR. **(B)** MD simulation ligand-protein interaction RMSF profile of quercetin-TYR. **(C)** MD simulation ligand-protein interaction RMSD profile of luteolin-TYR. **(D)** MD simulation ligand-protein interaction RMSF profile of luteolin-TYR. **(E)** MD simulation ligand-protein interaction RMSD profile of myricetin-TYR. **(F)** MD simulation ligand-protein interaction RMSF profile of myricetin-TYR. **(G)** MD simulation ligand-protein interaction RMSD profile of apigenin-TYR. **(H)** MD simulation ligand-protein interaction RMSF profile of apigenin-TYR.

Based on the results of molecular docking, we selected small molecules quercetin, luteolin, myricetin, and apigenin with strong binding energy and receptor TYR for MD simulation to further verify the dynamic characteristics of their interactions, including RMSD, RMSF, Cour-SR and LJ-SR interaction energy to describe the stability of ligand-receptor in 0–50 ns, as shown in Figure 8, Supplementary Figure S8 and Table 1. The RMSD of quercetin, luteolin, myricetin and apigenin were binding to TYR remained stable below 5 nm, indicating that they were in a consistent state during the simulation. In the presence of TYR protein molecules, it can be seen from the RMSD peak diagram that quercetin, luteolin, myricetin and apigenin fluctuate smoothly, indicating that they can be combined with TYR stably, and the overall carbon skeleton fluctuation state of TYR is also stable (Figures 8B,D,F,H). RMSF represents the degree of freedom of each atom in the molecule. RMSF analysis of the four sets of trajectories shows that there is no significant fluctuation in the key residues of TYR. The RMSF values of the key residues in the active pocket were analyzed. The RMSF values of the key residues of quercetin, luteolin, myricetin, and apigenin interacting with TYR were at a relatively low level, with an average value of about 0.5 nm, indicating that the key residues lost flexibility due to the binding of TYR to small molecule ligands, making it stable (Figures 8B,D,F,H). In addition, to quantify the strength of the interaction between quercetin, luteolin, myricetin, and apigenin with TYR, Cour-SR, and LJ-SR were used to calculate the nonbonded interaction energy. As shown in Table 1 and Supplementary Figure S8, the total average interaction energy (kJ/mol) (Average Cour-SR interaction energy (kJ/mol) + Average LJ-SR interaction energy (kJ/mol) from high to low are myricetin-TYR, apigenin-TYR, luteolin-TYR and quercetin-TYR, respectively. Therefore, myricetin is more likely to bind to TYR receptors and bind more stably.

**TABLE 1:** The average protein-ligand interaction energy of quercetin, luteolin, myricetin, apigenin and TYR.

Interaction energy Ligand-protein	Average Cour-SR interaction energy (kJ/mol)	Average LJ-SR interaction energy (kJ/mol)	Total average interaction energy (kJ/mol)
quercetin-TYR	−67165.9	−3741.26	−70907.16
luteolin-TYR	−67673.1	−3514.27	−71187.37
myricetin-TYR	−67967.8	−3607.14	−71574.94
apigenin-TYR	−67860	−3633	−71493

## 4 Discussion

Compared with synthetic raw materials, natural extracts in traditional Chinese medicine are known for their multiple components, multiple targets, and versatility (Smit et al., 2009; Zhao et al., 2022). They can inhibit the activity of TYR in melanin synthesis, reduce the number of melanin transporters, and accelerate skin metabolism. Its advantages include minimal side effects and almost no skin irritation. After the product is used, the residual substance can be naturally degraded without polluting the environment. Traditional depigmentation agents, such as hydroquinone, corticosteroids, and kojic acid are very effective, but long-term exposure can cause some safety problems (e.g., time loss, atrophy, canceration, and other local or systemic side effects) (de la Caridad Hernandez et al., 2024). Numerous studies have shown that extracts from various natural sources exhibit significant efficacy in skin whitening and anti-aging activities (Kim et al., 2018). Currently, the natural raw materials applied and the biological raw materials extracted encompass a wide range of animals and plants. Among them, plants and their extracted components account for a large proportion, animals and their extracted components are mainly larger mammals, and small animals, including insects, account for a smaller proportion (Joyce et al., 2021). In a few insect studies, it has been reported that silkworm extracts and *Tenebrio molitor* larvae extracts have antioxidant activity and inhibitory effects on TYR (Kim et al., 2015; Kim et al., 2018; Manosroi et al., 2010). At present, the research on *Blaps rhynchopetera* Fairmaire mainly focuses on classification, component identification, and pharmacological mechanism, which is an insect that has received limited attention in the existing literature. In traditional applications, many beneficial drug effects have been noted, including anti-tumor and regulation of immune function, but its pharmacological mechanism is still unclear (Guang-ming, 2009; Xiao et al., 2018; Yang et al., 2019). In this study, we focused on evaluating the effect of EBR on TYR inhibitory activity, to determine whether EBR can inhibit the formation of melanin and become a cosmetic with whitening effect.

For the determination of melanin content and TYR inhibiting activity, *in vitro* and *in vivo* studies showed that the EEBR showed greater activity than the WEBR, this may be due to the polarity of ethanol being stronger than water, and can better dissolve the compound (Sun et al., 2015). In addition, we found that EBR and arbutin have better tyrosinase activity in cell experiments than *in vitro* experiments, which may be attributed to the simplified *in vitro* system and the complex cellular environment. The specific reasons need to be verified by subsequent experiments. We also determined the content of phenols and flavonoids in the EBR. It was found that the content of phenols and flavonoids in the EEBR was more significant (Figures 3A–C), which may be attributed to the fact that the compounds in *B. rhynchopetera* Fairmaire were more soluble in ethanol and insoluble in water. Therefore, we used UHPLC-Q-TOF MS to detect the compounds present in the EBR, focusing on phenols and flavonoids, as these compounds may be potential TYR inhibitors that can improve melanin deposition. It is well known that phenols and flavonoids are natural secondary metabolites have been shown to have potential uses, including antioxidant, antibacterial, anti-inflammatory and other effects, and can resist ultraviolet radiation to avoid oxidative stress and anticancer (Rensing, 2018). Many phenols and flavonoids, such as chalcone, resveratrol, and coumarin have been reported to inhibit TYR activity, which in turn inhibits melanin production (Chang, 2009; El-Nashar et al., 2021; Pillaiyar et al., 2017). We found phenols and flavonoids in the EBR. It is speculated that *B. rhynchopetera* Fairmaire may synthesize or absorb phenols and flavonoids from the surrounding environment or from fed plants, thereby inhibiting TYR activity. This hypothesis needs to be confirmed by further studies. In insects, TYR is also one of the key enzymes in the molting process (Likhitwitayawuid, 2008). The study of inhibitors of this enzyme may lead to the development of new skin-whitening agents, and anti-browning substances.

GO and KEGG analysis of the intersection genes of compounds and targets showed that the EBR affected phosphorylation metabolism, negative regulation of apoptosis, and participated in pathways in cancer, PI3K-Akt signaling pathway, MAPK signaling pathway, and EGFR tyrosine kinase inhibitor resistance and were ranked at the top. These signaling pathways have been reported to be related to the TYR signaling pathway (Moon et al., 2023). In these processes, the MITF in melanocytes activates signaling pathways by interacting with receptors on the melanin membrane, including the above pathways, which not only regulate the proliferation and survival of melanocytes but also regulate the expression of TYR (Hu et al., 2021; Lv et al., 2020; Pillaiyar et al., 2018). For the PI3K-Akt signaling pathway, PI3K activates AKT, and activated AKT induces GSK3β phosphorylation. GSK3β is involved in PA regulation of melanin formation and inhibits melanin formation (Liu et al., 2020). EGFR is a key player in cell growth, wound healing, and maintenance of skin homeostasis, controlling skin pigmentation (Xu P. et al., 2024). The above results indicate that the EBR inhibits TYR by targeting these signaling pathways.

Next, we conducted a network pharmacology and molecular docking analysis of the phenol and flavonoid compounds found and further explored which compound has the function of inhibiting TYR activity and improving melanin deposition. A ‘component-target-disease’ network was constructed for the potential whitening component targets of *B. rhynchopetera* Fairmaire, and 225 nodes and 933 interacting edges were calculated. At the same time, the top four key components of quercetin, myricetin, luteolin, and apigenin were screened out. After the construction of the PPI network, TYR, AKT1, and EGFR, three key genes involved in whitening, were identified. We then performed molecular docking of these compounds and genes, finding that they exhibited good binding energy, with values less than −7 kJ/mol. In addition, MD simulation indicated that myricetin is more likely to bind to TYR receptors and binds more stably. Studies have indicated that *Bubonium imbricatum* exhibits significant TYR inhibitory activity, with the abundant presence of luteolin, apigenin 7-glucoside, and apigenin in the extracts likely contributing significantly to this activity (Aghraz et al., 2018). Myricetin and quercetin have been reported to have various degrees of inhibitory activity toward TYR (Khan et al., 2011; Matsuda et al., 1996; Shin et al., 1998). TYR hydroxylates *L*-tyrosine to form *L*-DOPA, which promotes the formation of melanin (Qian et al., 2020). AKT1 is involved in EGFR tyrosine kinase inhibition resistance pathway, apoptosis and neuroprotection (Zhao et al., 2024). AKT1 pathway plays an important role in skin wound healing and delaying and preventing cell senescence (Somanath et al., 2008), indicating that *B. rhynchopetera* Fairmaire can regulate these targets to treat skin blackening, improve melanin deposition and delay skin aging. Platelet-derived growth factor (PDGF) in serum is essential for the induction of melanogenesis. It is a TYR kinase on the cell surface, which induces the cell surface receptor TYR kinase of EGFR, forms a heterodimer with PDGFR, activates the downstream melanogenesis/carcinogenic signaling pathway involving PAK1 (P21 (RAC1) activated kinase 1), and finally activates MITF necessary for the expression of TYR and its related protein (TRP) gene (Maruta et al., 2017).

In summary, these findings highlight the remarkable antioxidant and TYR inhibitory properties of *B. rhynchopetera* Fairmaire, suggesting its potential whitening effects. Further studies, including *in vivo* experiments and clinical trials, are necessary to validate and explore its therapeutic applications.

## 5 Conclusion

In this study, we identified the primary bioactive components of the EBR, including a range of polyphenols and flavonoids. Among them, based on integrated metabolomics and network pharmacology, our findings suggest that myricetin, luteolin, apigenin, and quercetin may be the main compounds responsible for the whitening activity of *B. rhynchopetera* Fairmaire. This work reveals that insect extracts may have broad application prospects in cosmetics preparation, and further clinical research is needed to explore the potential application of EBR as a whitening product.

## Data Availability

The original contributions presented in the study are included in the article/Supplementary Material, further inquiries can be directed to the corresponding authors.

## Abbreviations

EBR, Extracts of Blaps rhynchopetera Fairmaire; WBRE, Water extract of Blaps rhynchopetera Fairmaire; EEBR, ethanol extract of Blaps rhynchopetera Fairmaire; ESI+, Positive electrospray ionization; ESI-, Negative electrospray ionization; TYR, Tyrosinase; MITF, Melanocyte-inducing transcription factor; EGFR, Epidermal growth factor receptor; IHC, Immunohistochemistry; UV, Ultraviolet; UVB, Ultraviolet radiation b; DMSO, dimethyl sulfoxide; RMSD, root-mean-square deviation; RMSF, root-mean-square fluctuation; MD, Molecular dynamics; PBS, Phosphate buffered saline; CCK-8, Cell counting kit-8.
