# Supplementary material for: Discovery of insect Blaps rhynchopetera Fairmaire extracts with anti-tyrosinase activity and anti-melanin deposition
Source: Front Pharmacol. 2025 Aug 20;16:1595534. doi: 10.3389/fphar.2025.1595534 (PMC12405244; doi:10.3389/fphar.2025.1595534)
Supplement: Supplementary file 1 [file Supplementaryfile1.docx]

**Supplementary**

**Primer table 1**

|  | Gene name | Forward primer | Reverse primer |
| --- | --- | --- | --- |
| B16F10 | TYR | 5’-GGCCAGCTTTCAGGCAGAGGT-3’ | 5’-TGGTG-CTTCATGGGCAAAATC-3’ |
|  | MITF | 5’-AAGTG-GTCTGCGGTGTCTCC-3’ | 5’-GTTGTTGGTAAA-GGTGATGG-3’ |
|  | GAPDH | 5’-CGT CCC GTA GAC AAA ATG GT-3’ | 5’-TTG ATG GCA ACA ATC TCC AC-3’ |
| guinea pig | TYR | 5’-GGCCAGCTTTCAGGCAGAGGT-3’ | 5’-TGGTGCTTCATG GGCAAAATC-3’ |
|  | MITF | 5’-CAGAGGCACCAGGTAAAGCA-3’ | 5’-GGATCCATCAAG CCCAAAAT-3’ |
|  | GAPDH | 5’-TGGAATCCTGTGGCATCCATGAAAC-3’ | 5’-TAAAACGCAGCTCAGTAACAGTCCG-3’ |

**
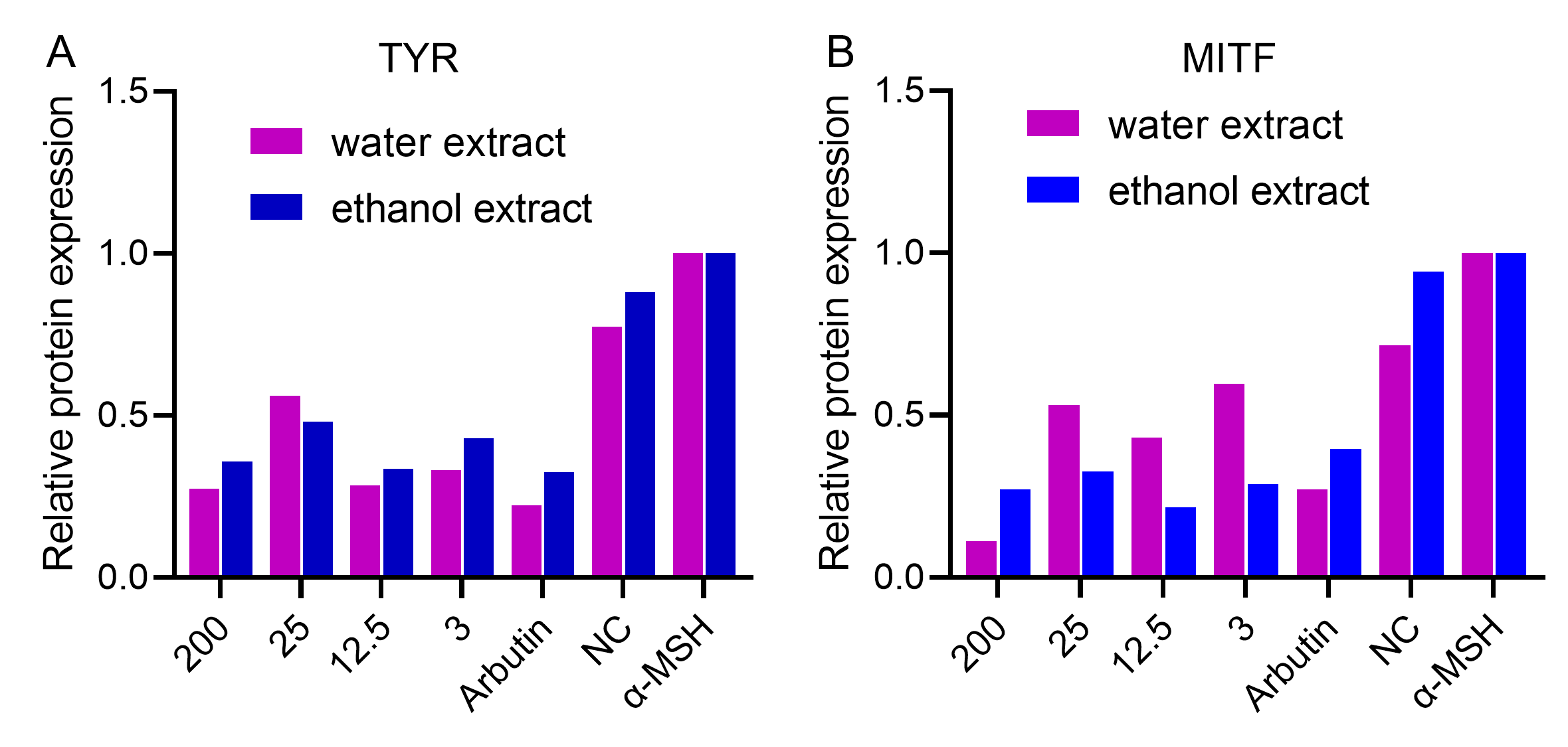
**

**Figure S1.** Image J analysis of TYR and MITF protein expression levels in the B16F10 cells for EBR in western blot experiments.

**Figure S2.** Image J analysis of TYR and MITF protein expression levels in the in the skin of guinea pigs for EBR in western blot experiments.

**
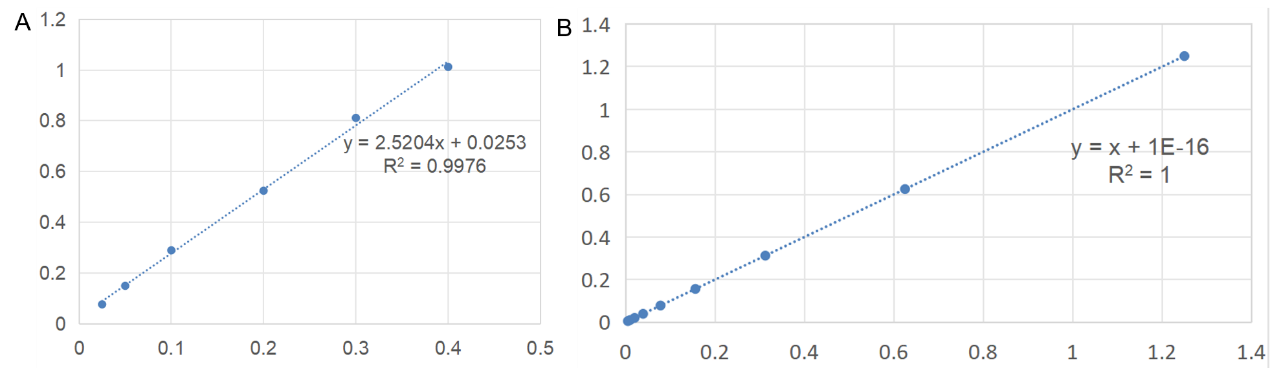
**

**Figure S3.** (A) Standard curve of total phenols content. (B) Standard curve of total flavonoids content.


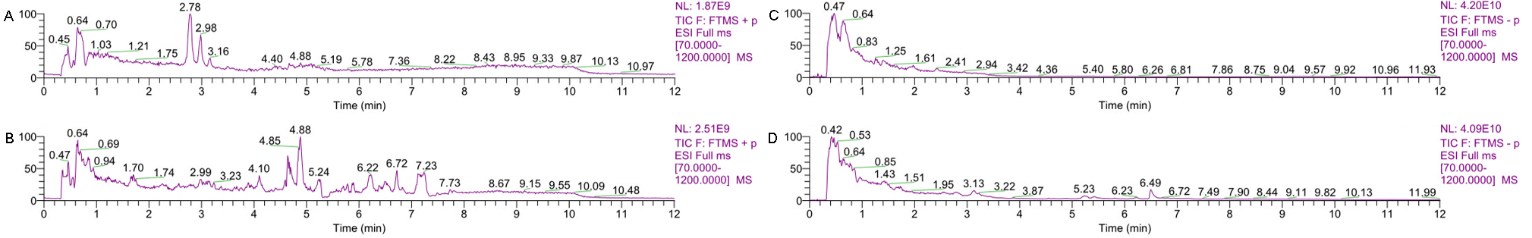


**Figure S4.** Total ion chromatogram (TIC) of water and ethanol extraction. (A) Positive ion mode of water extraction. (B) Positive ion mode of ethanol extraction. (C) Negative ion mode of water extraction. (D) Negative ion mode of ethanol extraction.


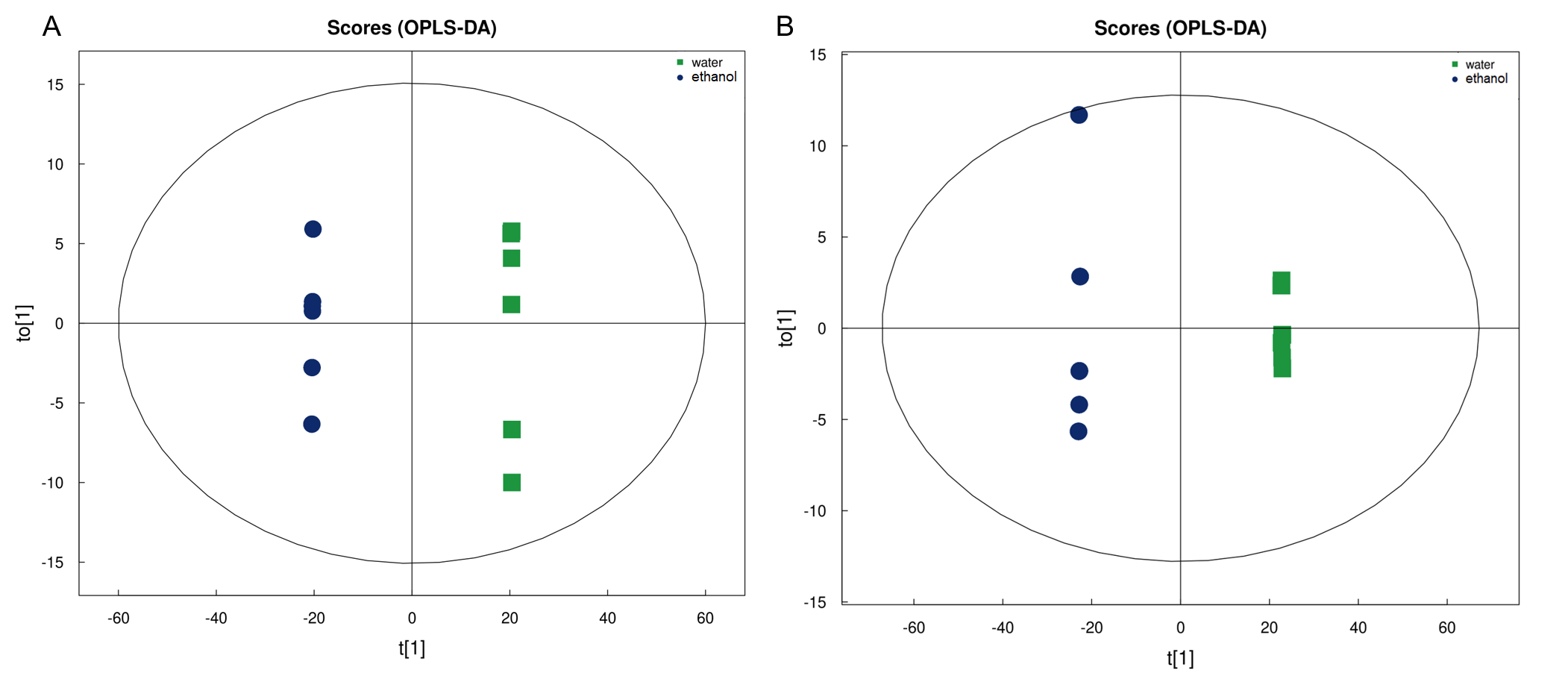


**Figure S5.** OPLS-DA analysis of water and ethanol extraction**.** (A) OPLS-DA of positive mode. (B) OPLS-DA of negative mode. t[1] represents principal component 1, to[1] represents principal component 2, and ellipse represents 95% confidence interval. The points of the same color represent the biological repetitions in the group, and the distribution of the points reflects the degree of difference between groups and within groups.

**Table 2**. The flavonoids and phenols in the extract of *B. rhynchopetera* were identified by UHPLC-TOF MS.

| Component name | RT(s) | m/z | Formula | KEGG | Adduct | Ppm |
| --- | --- | --- | --- | --- | --- | --- |
| flvonoids |  |  |  |  |  |  |
| 5,6,7,3',4'-pentamethoxyflavone | 229.2075 | 373.13963 | C_20_H_20_O_7_ | C10186 | [M+H]^+^ | 0.627927921 |
| 7,4'-dihydroxyflavone | 84.1634 | 255.06541 | C_15_H_10_O_4_ | C12123 | [M+H]^+^ | 1.265040607 |
| Acacetin | 36.4359 | 285.07596 | C_16_H_12_O_5_ | C01470 | [M+H]^+^ | 0.642708197 |
| Amentoflavone | 90.4441 | 539.09636 | C_30_H_18_O_10_ | C10018 | [M+H]^+^ | 1.56630652 |
| Apigenin | 161.726 | 271.0603 | C_15_H_10_O_5_ | C01477 | [M+H]^+^ | 1.062878637 |
| Astragalin | 205.311 | 471.08983 | C_21_H_20_O_11_ | C12249 | [M+Na]^+^ | 0.003406454 |
| Baicalein trimethyl ether | 35.0542 | 313.10727 | C_18_H_16_O_5_ | C10024 | [M+H]^+^ | 0.266534497 |
| Epigallocatechin gallate | 21.6268 | 459.10584 | C_22_H_18_O_11_ | C09731 | [M+H]^+^ | 2.505254983 |
| Flavone | 59.31425 | 223.08279 | C_15_H_10_O_2_ | C10043 | [M+H]^+^ | 1.14994482 |
| Gardenin b | 467.8045 | 359.12486 | C_19_H_18_O_7_ | C15109 | [M+H]^+^ | 1.138041443 |
| Hinokiflavone | 333.5635 | 539.10151 | C_30_H_18_O_10_ | C10057 | [M+H]^+^ | 0.461889594 |
| Icariin | 188.298 | 531.16313 | C_33_H_40_O_15_ | C17555 | [M+H-C6H10O4]^+^ | 0.223862464 |
| Keracyanin | 23.1396 | 595.15914 | C_27_H_31_O_15_ | C08620 | [M]^+^ | 1.508070993 |
| Luteolin | 205.278 | 287.05527 | C_15_H_10_O_6_ | C01514 | [M+H]^+^ | 0.342151498 |
| Myricetin | 298.374 | 319.06032 | C_15_H_10_O_8_ | C10107 | [M+H]^+^ | 1.371291094 |
| Narirutin | 21.2166 | 419.1129 | C_27_H_32_O_14_ | C09793 | [M+H-C_6_H_10_O_5_]^+^ | 0.273172936 |
| Nevadensin | 100.4895 | 345.09541 | C_18_H_16_O_7_ | C10111 | [M+H]^+^ | 0.980677889 |
| Nobiletin | 604.081 | 403.11572 | C_21_H_22_O_8_ | C10112 | [M+H]^+^ | 1.605518359 |
| Quercetin | 104.954 | 303.0267 | C_15_H_10_O_7_ | C00389 | [M+H]^+^ | 0.107403178 |
| Schaftoside | 166.131 | 547.15872 | C_26_H_28_O_14_ | C10181 | [M+H-H_2_O]^+^ | 2.405783567 |
| Spinosine | 23.4567 | 609.17342 | C_28_H_32_O_15_ | C17834 | [M+H]^+^ | 0.666717965 |
| Tangeretin | 21.8263 | 395.11262 | C_20_H_20_O_7_ | C10190 | [M+Na]^+^ | 0.743398525 |
| Tangeritin | 218.1605 | 395.12167 | C_20_H_20_O_7_ | C10190 | [M+Na]^+^ | 1.8359199 |
| Vitexin | 247.287 | 433.10379 | C_21_H_20_O_10_ | C01460 | [M+H]^+^ | 0.349818643 |
| (-)-catechin | 37.4976 | 187.03939 | C_15_H_14_O_6_ | C14079 | [M-H-C_4_H_6_O_3_]^-^ | 3.696919329 |
| (-)-epicatechin | 232.38 | 289.07203 | C_15_H_14_O_6_ | C09727 | [M-H]^-^ | 0.227596344 |
| (+)-catechin | 184.8265 | 245.08232 | C_15_H_14_O_6_ | C06562 | [M-H-CO_2_]^-^ | 0.850742337 |
| 3',5'-dimethoxy-3,5,7,4'-tetrahydroxyflavone | 29.2884 | 345.06157 | C_17_H_14_O_8_ | C11620 | [M-H]^-^ | 0.864107301 |
| 6-hydroxyflavanone | 38.5705 | 221.08199 | C_15_H_12_O_3_ | C14221 | [M-H-H_2_O]^-^ | 0.058041457 |
| 7-hydroxyflavanone | 191.4745 | 479.13505 | C_15_H_12_O_3_ | C14290 | [2M-H]^-^ | 0.472977697 |
| Apigenin 7-glucoside | 152.4495 | 431.09837 | C_21_H_20_O_10_ | C04608 | [M-H]^-^ | 0.202641997 |
| Astilbin | 21.77105 | 449.12412 | C_21_H_22_O_11_ | C17449 | [M-H]^-^ | 0.62050127 |
| Chrysin | 35.16405 | 253.05071 | C_15_H_10_O_4_ | C10028 | [M-H]^-^ | 0.490462905 |
| Cyanidin 3,5-diglucoside | 20.6145 | 627.15043 | C_27_H_31_O_16_ | C08639 | [M-2H+H_2_O]^-^ | 1.281690016 |
| Eriocitrin | 178.694 | 595.18322 | C2_7_H_32_O_15_ | C09732 | [M-H]^-^ | 3.879849773 |
| Eriodictyol 7-o-neohesperidoside | 109.518 | 459.09324 | C_27_H_32_O_15_ | C09805 | [M-H-C_8_H_8_O_2_]^-^ | 0.643198121 |
| Farrerol | 44.2116 | 179.03499 | C_17_H_16_O_5_ | C09734 | [M-H-C_8_H_8_O]^-^ | 0.356270229 |
| Fustin | 31.3002 | 287.05617 | C_15_H_12_O_6_ | C01378 | [M-H]^-^ | 0.370352438 |
| Ginkgetin | 55.866 | 565.11431 | C_32_H_22_O_10_ | C10048 | [M-H]^-^ | 0.844465269 |
| Hesperetin | 48.1985 | 283.06135 | C_16_H_14_O_6_ | C01709 | [M-H-H_2_O]^-^ | 0.136611344 |
| Isoorientin | 46.1608 | 429.0825 | C_21_H_20_O_11_ | C01821 | [M-H-H_2_O]^-^ | 0.06642377 |
| Isosakuranetin | 26.94935 | 285.07683 | C_16_H_14_O_5_ | C05334 | [M-H]^-^ | 0.155545722 |
| Morin | 188.018 | 301.05668 | C_15_H_10_O_7_ | C10105 | [M-H]^-^ | 0.091604343 |
| Naringenin | 30.8018 | 271.0613 | C_15_H_12_O_5_ | C00509 | [M-H]^-^ | 0.532496502 |
| Neohesperidin | 163.705 | 301.07199 | C_28_H_34_O_15_ | C09806 | [M-H-C_12_H_20_O_9_]^-^ | 1.314753775 |
| Plantaginin | 186.8435 | 447.09311 | C_21_H_20_O_11_ | C17056 | [M-H]^-^ | 0.729830496 |
| Procyanidin b2 | 127.659 | 577.11463 | C_30_H_26_O_12_ | C17639 | [M-H]^-^ | 2.453892649 |
| Quercetin 3'-methyl ether | 169.017 | 315.07244 | C_16_H_12_O_7_ | C10084 | [M-H]- | 0.345276299 |
| Quercitrin | 315.542 | 447.114 | C_21_H_20_O1_1_ | C01750 | [M-H]^-^ | 0.791642335 |
| Rhamnetin | 25.9294 | 315.05102 | C_16_H_12_O_7_ | C10176 | [M-H]^-^ | 0.693395941 |
| Skullcapflavone ii | 21.7171 | 373.09295 | C_19_H_18_O_8_ | C10183 | [M-H]^-^ | 1.026106205 |
| Swertisin | 163.07 | 325.07224 | C_22_H_22_O_10_ | C17835 | [M-H-C_4_H_8_O_4_]^-^ | 1.128276692 |
| Tricin methyl ether | 221.366 | 343.08252 | C_18_H_16_O_7_ | C19807 | [M-H]^-^ | 1.960118237 |
| phenols |  |  |  |  |  |  |
| 4-hydroxymandelonitrile | 95.7961 | 150.05501 | C_8_H_7_NO_2_ | C03742 | [M+H]^+^ | 0.659168639 |
| Acetaminophen | 38.9211 | 152.07071 | C_8_H_9_NO_2_ | C06804 | [M+H]^+^ | 1.541783228 |
| Dl-4-hydroxy-3-methoxymandelic acid | 38.7026 | 181.04967 | C_9_H_10_O_5_ | C05584 | [M+H-H_2_O]^+^ | 2.239025751 |
| Dl-normetanephrine | 9.98075 | 206.10368 | C_9_H_13_NO_3_ | C05589 | [M+Na]^+^ | 1.270516741 |
| Dopamine | 364.086 | 154.08366 | C_8_H_11_NO_2_ | C03758 | [M+H]^+^ | 0.344484961 |
| Epinephrine | 325.878 | 184.09463 | C_9_H_13_NO_3_ | C00788 | [M+H]^+^ | 1.234817463 |
| Feruloyl tyramine | 32.2745 | 336.12283 | C_18_H_19_NO_4_ | C02717 | [M+Na]^+^ | 0.221668454 |
| Isoproterenol | 347.1475 | 194.11766 | C_11_H_17_NO_3_ | C07056 | [M+H-H_2_O]^+^ | 0.627997528 |
| 1,2,3-benzenetriol | 47.81775 | 125.02448 | C_6_H_6_O_3_ | C01108 | [M-H]^-^ | 0.174702514 |
| 3-methylcatechol | 44.2856 | 123.04521 | C_7_H_8_O_2_ | C02923 | [M-H]^-^ | 0.108466209 |
| 3-nitrophenol | 86.68575 | 108.0217 | C_6_H_5_NO_3_ | C14418 | [M-H-NO]^-^ | 0.885350454 |
| 3,4-dihydroxymandelic acid | 315.197 | 367.07921 | C_8_H_8_O_5_ | C05580 | [2M-H]^-^ | 3.354304658 |
| 3,4-dihydroxyphenylacetic acid | 339.9965 | 166.99865 | C_8_H_8_O_4_ | C01161 | [M-H]^-^ | 0.369669651 |
| 4-methylcatechol | 185.319 | 123.04519 | C_7_H_8_O_2_ | C06730 | [M-H]^-^ | 0.168662901 |
| 4,6-dinitro-o-cresol | 86.35875 | 137.02447 | C_7_H_6_N_2_O_5_ | C18653 | [M-H-N_2_O_2_]^-^ | 0.149950048 |
| Coniferyl aldehyde | 23.9546 | 177.05564 | C_10_H_10_O_3_ | C02666 | [M-H]^-^ | 0.46314179 |
| Dihydroconiferyl alcohol | 139.688 | 181.08709 | C_10_H_14_O_3_ | C10448 | [M-H]^-^ | 0.229372597 |
| Dl-3,4-dihydroxyphenyl glycol | 48.8156 | 151.03999 | C_8_H_10_O_4_ | C05576 | [M-H-H_2_O]^-^ | 0.64607371 |
| DL-Vanillylmandelic acid | 153.117 | 197.03176 | C_9_H_10_O_5_ | C05584 | [M-H]^-^ | 0.444973242 |
| Dobutamine | 322.24 | 300.18188 | C_18_H_23_NO_3_ | C06967 | [M-H]^-^ | 1.094324361 |
| Homovanillic acid | 128.017 | 181.05037 | C_9_H_10_O_4_ | C05582 | [M-H]^-^ | 1.776548013 |
| Hydroquinone | 130.3345 | 109.02911 | C_6_H_6_O_2_ | C00530 | [M-H]^-^ | 3.917323301 |
| Orcinol | 24.6116 | 123.00872 | C_7_H_8_O_2_ | C00727 | [M-H]^-^ | 0.103414561 |
| Phenol | 38.3683 | 93.03459 | C_6_H_6_O | C00146 | [M-H]^-^ | 0.402388293 |
| Pyrocatechol | 39.47795 | 109.02951 | C_6_H_6_O_2_ | C00090 | [M-H]^-^ | 0.161040483 |
| Resorcinol | 698.689 | 109.04076 | C_6_H_6_O_2_ | C01751 | [M-H]^-^ | 0.663679311 |
| Sinapyl alcohol | 190.155 | 209.04833 | C_11_H_14_O_4_ | C02325 | [M-H]^-^ | 3.242472044 |
| Trans-3,5-dimethoxy-4-hydroxycinnamaldehyde | 38.06435 | 207.06631 | C_11_H_12_O_4_ | C05610 | [M-H]^-^ | 0.055830979 |


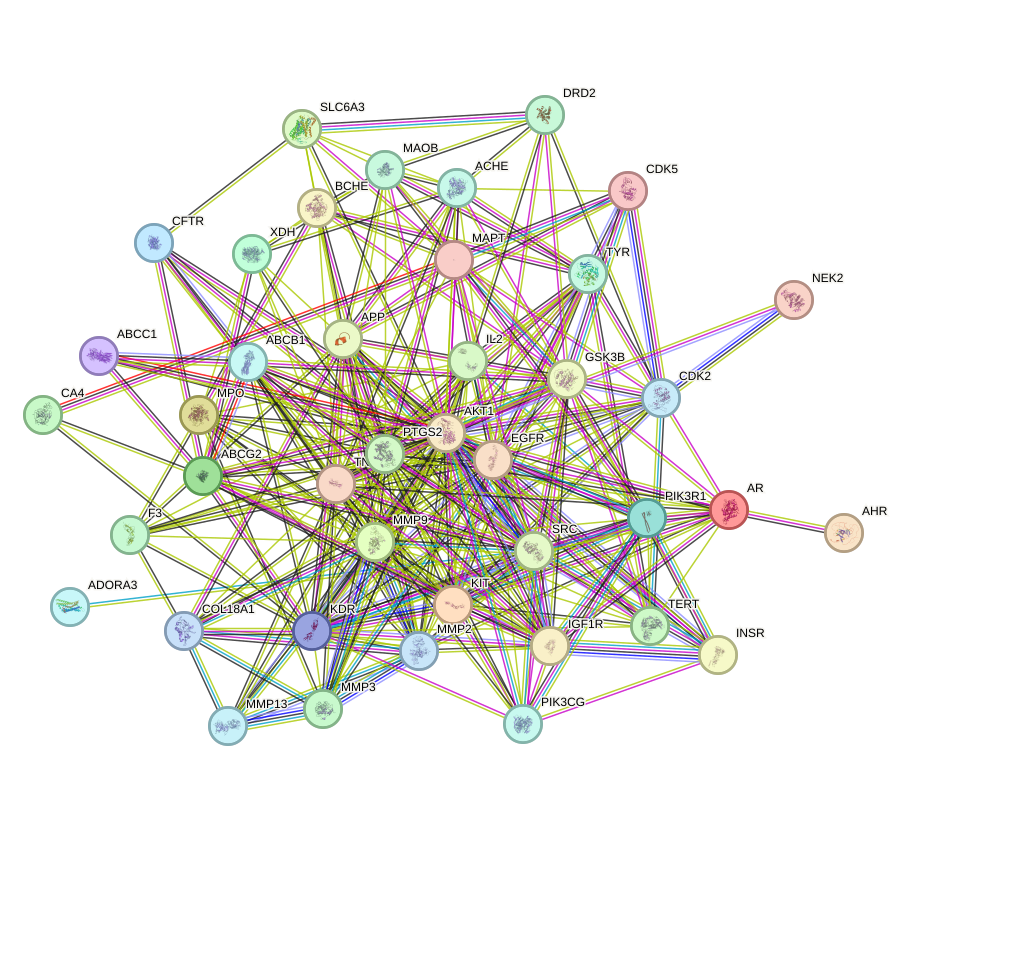


**Figure S6**. PPI network diagram of B. rhynchopetera and melanin deposition intersection gene.


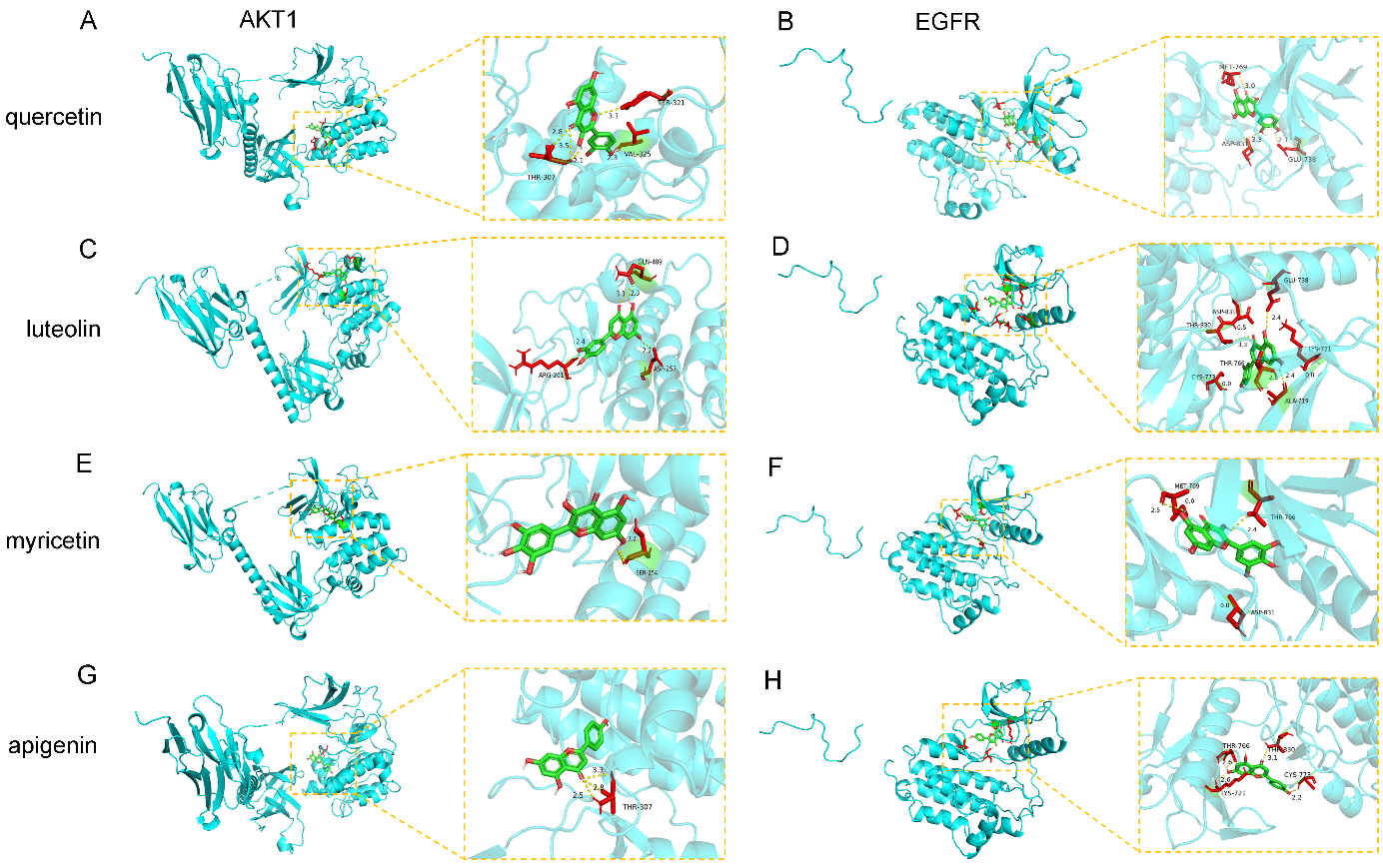


**Figure S7**. Visual analysis of molecular docking. (A) quercetin-AKT1; (B) quercetin-EGFR; (C) luteolin-AKT1; (D) luteolin-EGFR; (E) myricetin-AKT1; (F) myricetin-EGFR; (G) apigenin-AKT1; (H) apigenin-EGFR.


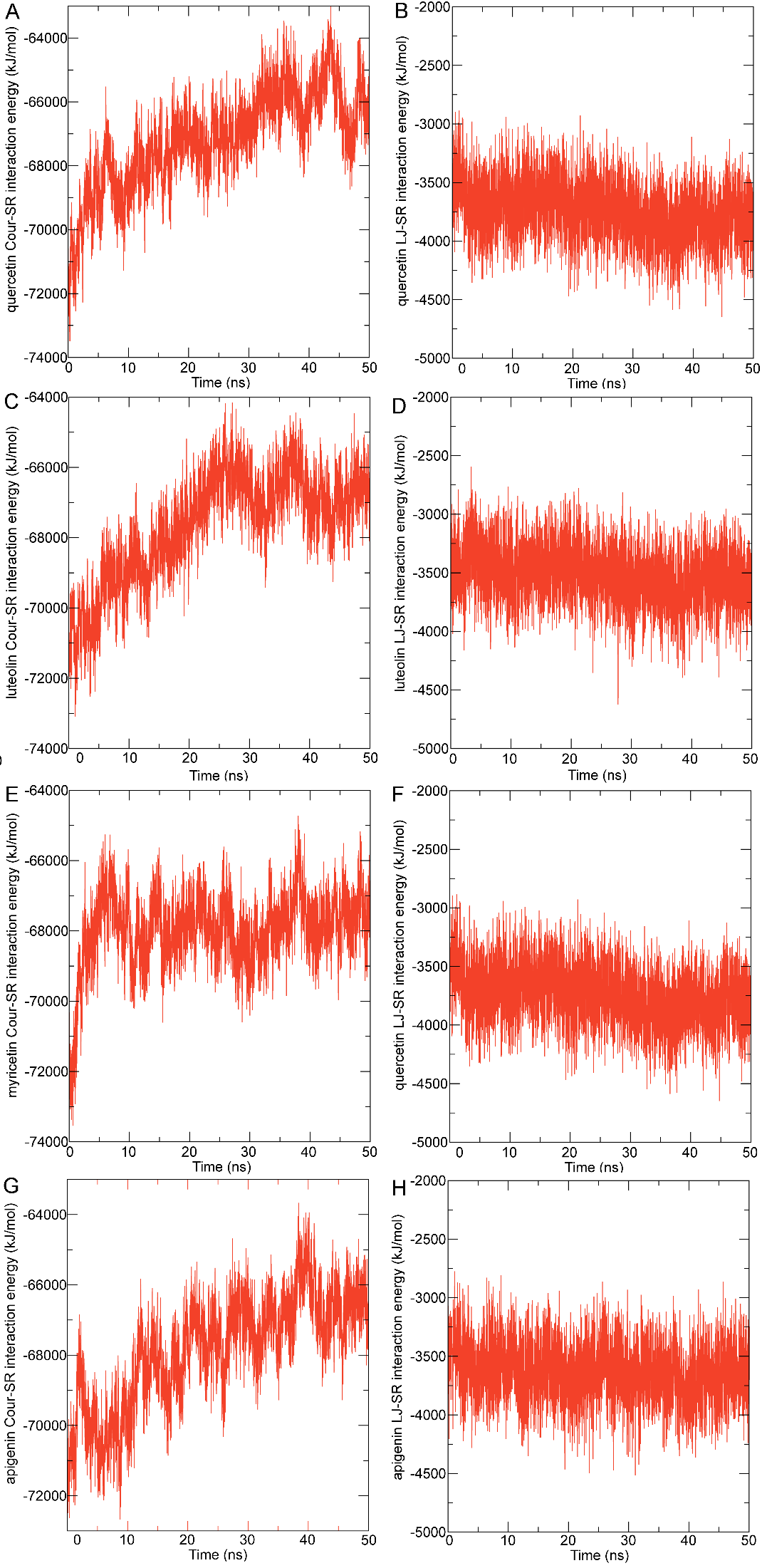


**Figure S8**. (A) short-range Coulombic (Cour-SR) interaction energy of quercetin-TYR. (B) short-range Lennard-Jones (LJ-SR) interaction energy of quercetin-TYR. (C) Cour-SR interaction energy of luteolin-TYR. (D) LJ-SR interaction energy of luteolin-TYR. (E) Cour-SR interaction energy of myricetin-TYR. (F) LJ-SR interaction energy of myricetin-TYR. (G) Cour-SR interaction energy of apigenin-TYR. (H) LJ-SR interaction energy of apigenin-TYR.
